# Pre-clinical studies comparing the anti-inflammatory potential of artemisinic compounds by targeting NFκB/TNF-α/NLRP3 and Nrf2/TRX pathways in Balb/C mice

**DOI:** 10.3389/fphar.2024.1352827

**Published:** 2024-06-07

**Authors:** Syeda Tayyaba Batool Kazmi, Humaira Fatima, Iffat Naz, Nosheen Kanwal, Ihsan-ul Haq

**Affiliations:** ^1^ Department of Pharmacy, Faculty of Biological Sciences, Quaid-i-Azam University, Islamabad, Pakistan; ^2^ Department of Biology, College of Science, Qassim University, Almolaydah, Buraydah, Saudi Arabia; ^3^ Department of Chemistry, College of Science, Qassim University, Almolaydah, Buraydah, Saudi Arabia

**Keywords:** antioxidant activity, antiinflammatory activity, inflammation, *in vivo* study, reactive oxygen species

## Abstract

Artemisinin, artemether, artesunate, and dihydroartemisinin are renowned for their antimalarial potential. The current study aims to repurpose the above-mentioned artemisinic compounds (ACs) by conducting an intercomparison to evaluate their antiinflammatory potential (AIP). In order to develop potential candidates for the evaluation of AIP of ACs (50 and 100 mg/kg BW), carbon tetrachloride (1ml/kg body weight (BW)) was administered intraperitoneally to BALB/c mice. Alterations in animal behavior were assessed weekly through tail suspension test, force swim test, open field test, Y-maze test, inverted screen analysis, and weight lifting test. Aberrations in hematological, serological, endogenous antioxidants, and oxidative stress marker profiles were assessed in all twelve groups. Histological alterations were read using hematoxylin and eosin staining. Levels of inflammatory markers including nuclear factor kappa B (NF-κB), tumor necrosis factor alpha (TNF-α), and nucleotide-binding oligomerization domain-like receptor protein 3 (NLRP3), were determined using immunohistochemical analysis (IHCA). Antioxidant markers i.e., nuclear factor erythroid-2-related factor (Nrf-2) and thioredoxin (TRX) were also quantified through IHCA. Comet assay was performed to quantify DNA damage. Oral administration of ACs to mice significantly alleviated the carbon tetrachloride induced inflammation in comparison with silymarin. Reduced levels of several inflammatory markers including nitric oxide, thiobarbituric acid reactive substances, interleukin-1 beta, NF-κB, TNF-α, and NLRP3, underscore the substantial AIP of ACs. IHCA depicted the revitalized percent relative expression of Nrf-2 and TRX in groups treated with ACs. Behavioral analysis revealed that ACs-treated groups significantly (p<0.05) attenuated the memory deficit, anxiety, and depressive-like behavior. Moreover, histopathological, hematological, serological, and endogenous antioxidant profiles indicated substantial AIP of ACs. Findings of comet assay further bolstered the compelling evidence as DNA damage was significantly (p<0.05) curbed down after ACs (100 mg/kg) treatment. All these outcomes implied that ACs exhibited AIP in a dose-dependent manner with maximal AIP imparted by artemisinin (100 mg/kg). This pre-clinical investigation avers the tremendous AIP of ACs targeting key molecular pathways. The current study divulges artemisinin as the most potent antiinflammatory agent among the tested compounds.

## 1 Introduction

Inflammation is one of the biological processes standing in the foreground of numerous pathological conditions. Any alteration in tissue integrity orchestrates inflammation to restore tissue homeostasis via various repair mechanisms (Mahmood et al., 2022). Uncontrolled amplification of these mechanisms can shift the paradigm from tissue repair to collateral damage because of excessive reactive oxygen species (ROS) production (Kim et al., 2013).

Excessive ROS generation disrupts the redox homeostasis, precipitates extracellular matrix breakdown, and wreaks inevitable biomolecular damage, leading to cellular necrosis. These necrotic cells release several extracellular and intracellular alarmins that trigger inflammatory cascades in seriatim (Mahmood et al., 2022; Wasti et al., 2023). Leveraging the carbon tetrachloride (CCl_4_) metabolism is a robust and widely recognized experimental model that is adept at generating ROS (Nasir et al., 2022).

Mitigating oxidative stress using plant-based compounds with antioxidant capabilities is a potential anti-inflammatory strategy. The use of plant-based drugs has surged due to their safety and potency compared to synthetic pharmaceuticals (Abdallah et al., 2023; Badshah et al., 2023). Medicinal extracts of *Artemisia annua,* sweet wormwood or Qinghao, have been in use for nearly 2000 years. Ge Hong, a Chinese physician, illustrated an unusual technique of soaking the fresh herb in water, wringing it out, and taking the juice. This unorthodox approach might have helped in yielding an emulsion with plant oils containing the water-insoluble artemisinin (ART), which was isolated later in 1972 (Karunajeewa, 2012). X-ray crystallography revealed that ART is a sesquiterpene trioxane lactone. This immediately led to the generation of functionally similar derivatives. Hence, dihydroartemisinin (DHA), artemether (ARTEM), and artesunate (ARTES) were created. Chemically, DHA is a lactol, whereas ARTEM and ARTES are the methyl ether and sodium hemisuccinyl ester of DHA (Jansen, 2010). Generally known as ‘artemisinic compounds’ (ACs), these derivatives can also be divided into first-generation endoperoxides, such as DHA, and second-generation endoperoxides, such as ARTEM and ARTES. These compounds have a peroxide bridge within their lactone ring that is crucial for their activity (Cheong et al., 2020).

These ACs exhibit pleiotropic characteristics with antimalarial, anticancer, and antioxidant potential. Previously, these compounds have been found to inhibit the activation of several inflammatory mediators, including interleukin-6, tumor necrosis factor-alpha (TNF-α), and nitric oxide (NO^•^) (Okorji et al., 2016; Wang et al., 2017). However, the precise mechanism of their activities remains elusive. The paucity of data encompassing all the facets ranging from preliminary to mechanistic studies, which are prerequisites in the logical hunt for the anti-inflammatory potential of these compounds, makes current analysis prudent. Moreover, none of the studies done before have scientifically compared these artemisinic compounds to identify the metabolite with maximum anti-inflammatory potential. The current study was designed to evaluate and compare the antiinflammatory activity potential of selected interlinked ACs. It is the first of its kind to demonstrate the modulation of thioredoxin (TRX) proteins as a pathway by which ACs exert their anti-inflammatory potential.

## 2 Materials and methods

### 2.1 Chemicals

ART, ARTEM, ARTES, DHA, and CCl_4_ were procured from Sigma-Aldrich (Germany). Nicotinamide adenine dinucleotide (NADH), formalin, alcohol, sulfosalicylic acid, and ethidium bromide were purchased from Merck-Schuchardt, United States. Phosphate buffer (PB), 5,5′-dithio-bis-(2-nitrobenzoic acid) (DTNB), zinc sulfate, and sulfosalicylic acid were purchased from Riedel-de-Haen, Germany. Ethylenediaminetetraacetic acid (EDTA), formalin, sodium carbonate (Na_2_CO_3_), sodium bicarbonate (NaHCO_3_), low melting point agarose (LMPA), normal melting point agarose (NMPA), Tris HCl, starch, sulphanilamide, p-nitrophenyl-D-glucopyranose, acetonitrile and phosphoric acid were purchased from Merck, KgaA (Darmstadt, Germany). Guaiacol, trichloroacetic acid (TCA), thiobarbituric acid (TBA), hydrogen peroxide (H_2_O_2_), acetic acid, phenazinemethosulphate, triton X-100, nicotinamide adenine dinucleotide (NADH), ethidium bromide, hematoxylin/eosin (H&E), carboxy methyl cellulose (CMC), secondary antibody, and xylene were acquired from Merck-Schuchardt, United States. Silymarin was purchased from Abbott Laboratories (Pakistan) Limited. Antibodies, including rabbit polyclonal nuclear factor erythroid-2-related factor (Nrf-2; SC-722), mouse monoclonal nuclear factor Kappa-light-chain-enhancer of activated B cells (NF-κB; SC-271908), rabbit polyclonal TRX (SC-20146), mouse monoclonal TNF-α (SC-52746), ABC Elite kit (SC-2018), and 3,3′-diaminobenzidine peroxidase (DAB; SC-216567), were purchased from Santa Cruz Biotechnology (Dallas, TX,United States). Rabbit monoclonal nucleotide-binding oligomerization domain-like receptor protein 3 (NLRP3; Cat.No.A5652) was purchased from Abclonal, Woburn, MA, United States. Unless otherwise stated, all chemicals were obtained from Sigma-Aldrich (United States).

### 2.2 Apparatus and equipment

An ultrasonicator (Sweep Zone Technology, United States), a microtome HM 550 (Thermo Scientific, United States), a light microscope (Olympus, Japan), a fluorescence microscope (Irmeco, Germany), a centrifuge (B. Bran, Germany), a freezer 9170 WB M (Dawlance, Pakistan), a Neubauer hemocytometer (Feinoptik, Germany), beakers, Erlenmeyer flasks, Petri dishes, a magnetic stirrer, a magnifying glass, a funnel, and micropipettes and Pasteur pipettes (Sartorius, France) were utilized in the study.

### 2.3 Animals

All experimentation was performed on 7-week-old BALB/c mice of both sexes weighing 25–30 g. The animals were retained in Bio-rad PVC cages under controlled environmental conditions (60 ± 10% relative humidity, 23 ± 2°C temperature, and 12 h light/dark cycle) with a standard laboratory diet and free access to water.

### 2.4 Animal ethical statement

Animal studies were performed in compliance with the guidelines of the National Institute of Health, Islamabad, Pakistan, and the Organization for Economic Co-operation and Development (OECD Test Guideline: 453) after approval by the Bioethical Committee of Quaid-i-Azam University, Islamabad in letter # BEC-FBS-QAU2019-144B (BEC-FBS-QAU2022-421).

### 2.5 *In vivo* acute toxicity assessment

The guidelines provided by the OECD (Test Guideline: 420) with slight modifications according to system suitability were followed for the acute toxicity assessment. According to the principles, potentially fatal doses or doses known to cause pain, toxicity, and suffering were avoided. The initial doses chosen for acute toxicity testing studies were 5 and 50 mg/kg (Baig et al., 2022b). All the dosing was done by oral gavage in a single dose. As per the guidelines, the maximum volume for administration did not exceed 1 mL/100 g of body weight. Healthy BALB/c mice, weighing approximately 25–30 g, were selected, marked individually for identification, divided into 10 groups (n = 6; three males and three females), and housed in Bio-rad PVC cages. Group I was kept untreated. Group II was named the vehicle control and was administered 10% DMSO in CMC. Groups III to VI were specified as ART, ARTEM, ARTES, and DHA-treated groups and administered with 5 mg/kg of the respective compounds, whereas groups VII to IX were treated with 50 mg/kg of the respective compounds. Prior to dosing, the mice were acclimatized to the laboratory conditions for 5–7 days. Observed parameters included changes in body weight, diarrhea, diuresis, ptosis, lethargy, ataxia, prostrate, lacrimation, coma, exophthalmos, changes in eyes, mucous membranes, fur, skin, behavioral alterations, rate of food consumption, frequency of urination, abnormal posture, respiration, tremors, convulsion, and gait. The onset and duration of toxic symptoms were recorded systematically for 48 h after dosing. The groups that received 5 mg/kg and 50 mg/kg ACs had no signs of toxicity. No mortality was observed as well. Therefore, rats in the third set of groups were administered with a higher dose, 100 mg/kg ACs, and observed as before. Mice were kept under observation for 2 weeks to allow for the observation of delayed toxicity. During this study, the body weights of the animals were recorded on days 1, 7, and 14.

### 2.6 Experimental design

Because no toxic symptoms or mortality were observed, the animals were randomly divided into twelve groups (n = 5) to conduct the anti-inflammatory study as per the previously illustrated protocol (Nasir et al., 2022).

### 2.7 Dose selection and preparation

High (HD; 100 mg/kg BW) and low (LD; 50 mg/kg BW) doses of the ACs and doses of silymarin (100 mg/kg) and CCl4 (1 mL/kg) were selected based on previous reports (Ma et al., 2015; Ferraz et al., 2021; Nasir et al., 2022). Stock suspensions for the predetermined doses of ART, ARTEM, ARTES, DHA, and silymarin were prepared using 10% (v/v) DMSO in CMC solution (0.075% w/v), whereas CCl_4_ (30%) was solubilized in olive oil. As per the OECD guidelines, 2 mL/100 g was specified as the maximum volume to be administered. Sterilized disposable oral gavages were utilized to administer the ACs and controls, whereas CCl_4_ was administered intraperitoneally. All drug suspensions were prepared immediately before dosing.

### 2.8 Animal grouping

After being marked from 1 to 60, mice were assigned to ventilated Bio-rad PVC cages under hygienic conditions at the primate facility of Quaid-i-Azam University Islamabad, Pakistan.

**Group I:** Normal control
**Group II:** Vehicle control
**Group III:** Positive control
**Group IV**: Disease control
**Group V**: ARTHD
**Group VI**: ARTLD
**Group VII**: ARTEMHD
**Group VIII**: ARTEMLD
**Group IX**: ARTESHD
**Group X**: ARTESLD
**Group XI**: DHAHD
**Group XII**: DHALD

Group I remained untreated and received only standard chow feed and water *ad libitum.* Group II received 10% (v/v) DMSO in CMC solution (0.075% w/v). Groups III–XII were treated with CCl_4_ on alternate days for 2 weeks to induce inflammation. After administering seven doses of CCl_4,_ animals were treated with the specified doses of ACs. Silymarin was used as the positive control.

**FIGURE 1:**
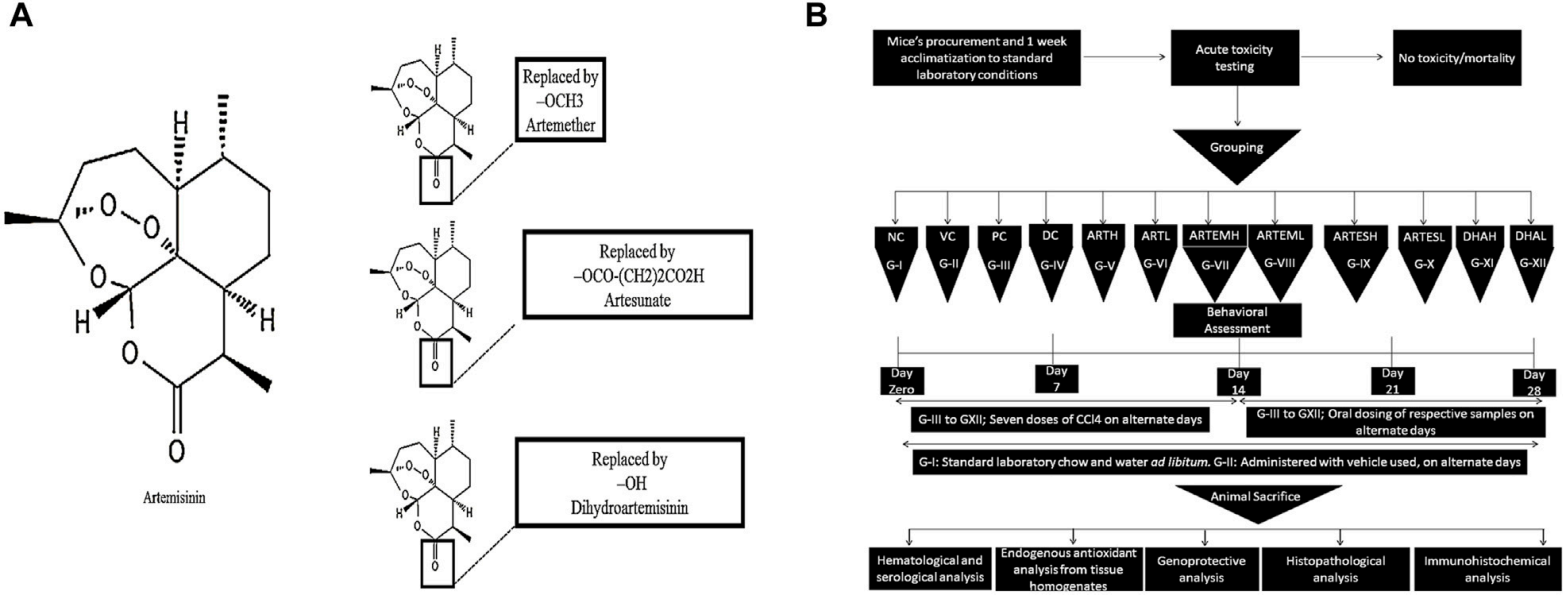
**(A)** Chemical structures of artemisinic compounds. Chemical structures have been sketched using PubChem Sketcher V2.4. **(B)** Schematic diagram of the methodology followed.

A total of seven doses of all the test moieties were administered on alternate days for 2 weeks. The non-toxic nature of the vehicle used was determined compared to that of the normal and the disease control for all the parameters tested. Figure 1 illustrates the chemical structures of the compounds used and the details of the study design.

### 2.9 Body weight measurement

The body weight of the experimental animals was measured every 2 weeks (Majid et al., 2019).

### 2.10 Behavioral determinations

Alterations in animal behavior were assessed on a weekly basis to evaluate the ameliorative effects of the compounds.

#### 2.10.1 Tail suspension test

The antidepressant effects of artemisinic compounds were evaluated through the tail suspension test (TST) (Khan et al., 2019). TST apparatus with the dimensions of 55 cm height (H) × 60 cm width (W) × 11.5 cm depth (D) was used for the study. Each animal was individually suspended on an aluminum bar (1 cm H × 1 cm W × 60 cm length (L)) for 6 min, inside its own rectangular compartment (55 cm H × 15 cm W × 11.5 cm D) to avoid its interaction with other test mice. An approximate distance of 20–25 cm was maintained between the tip of the animals’ nose and the floor of the apparatus. The duration of immobility was calculated during the last 5 min. Animals were considered immobile when they became inactive and were entirely motionless.

#### 2.10.2 Force swim test

Force swim test (FST) was also utilized as a model of depressive-like behavior (Yankelevitch-Yahav et al., 2015). Each animal was placed into the transparent Plexiglas cylindrical tanks (30 cm H × 20 cm diameter (d)) with a water level of 15 cm (24°C ± 1°C) for 6 min. The time during which the animal ceased to escape or floated motionlessly was counted as immobility duration in the last 4 min.

#### 2.10.3 Open-field test

Anxiety-like behavior was measured through the Open-field test (OFT) (Khan et al., 2019). A square-shaped open field apparatus with dimensions of 50 cm L × 50 cm W × 40 cm H with four central squares and 16 small squares was utilized in the study. This apparatus was placed in a soundproof room with a 60 W bulb lighted exactly above the center of the apparatus. Each animal was placed individually in the center, and a video recording was made for 5 min to evaluate the parameters like number of line crossings, time spent in the center, and number of rearings.

#### 2.10.4 Y-maze test

A polystyrene Y-maze apparatus with three perpendicular arms (50 cm L × 20 cm H × 10 cm W) was utilized in the study (Miedel et al., 2017). Each mouse was individually allowed to move freely at the center of the apparatus for 8 min. Arm entries were recorded through a video camera for three consecutive sessions. Successive entry of the animal into three arms in overlapping triplet sets was considered spontaneous alternation, which was calculated in percentage. Herein, a high percentage represents enhanced cognitive performance.

#### 2.10.5 Inverted screen test

Kondziela’s inverted screen test was performed to evaluate muscle coordination (Ullah et al., 2020). Each mouse was individually placed in the center of a wire mesh screen that was then held in an inverted position for 60 s. The time taken by the animal to fall off was recorded using a stopwatch as escape latency. The screen was held at a height of 40–50 cm above a padded surface to avoid any injury.

#### 2.10.6 Weight-lifting test

Anxiety-like behavior was measured through the open-field test (OFT) (Khan et al., 2019). Each mouse was individually lowered down to grip the first weight for 3 s. In case of failure to hold the first weight for 3 consecutive seconds, the activity was repeated in triplicate, and the maximum time for the heaviest weight lifted was assigned. In the cases when the animal grasped the weight for 3 consecutive seconds, the next heaviest weight was tried. The total score was calculated as the product of linked weights in the heaviest chain multiplied by the time the weight was held. If the heaviest weight was dropped before 3 s, an appropriate intermediate value was calculated.

### 2.11 Collection of organs, blood, and serum samples

After the 4-week treatment, mice were kept unfed with free access to water only for 24 h. Then, the animals were anesthetized using chloroform. Blood samples were drawn with a 5 mL Becton Dickinson (BD) syringe (25-gauge × 1-inch needle size) via cardiac puncture and immediately transferred to previously labeled BD vacationers for serological and hematological analysis. The serum was separated employing centrifugation and stored at −80°C for biochemical analysis. The anesthetized animals were then sacrificed by cervical dislocation, and organs, including the brain, heart, liver, and kidneys, were excised, washed, and preserved at −80°C for further studies. For histopathological and immunohistochemical (IHC) examinations, organs were immediately transferred to a fixative solution of 10% formalin.

### 2.12 Hematological studies

A Neubauer hemocytometer was utilized to count platelets, white blood cells (WBCs), and red blood cells (RBCs). Hemoglobin (Hb) content was measured using Sahli’s hemoglobinometer. Percent hematocrit (%HCT) was estimated using the microhematocrit method. The modified Westergren method was followed to measure the erythrocyte sedimentation rate (ESR) (Attia et al., 2016).

### 2.13 Serological analysis

Biochemical markers, including alanine transaminase (ALT), aspartate transferase (AST), albumin, urea, and creatinine, were estimated in serum samples using conventional AMP diagnostic kits (Stattogger Strasse 31b 8,045 Graz, Austria). Cardiac enzymes, including creatine kinase (CK) and lactate dehydrogenase (LDH), were measured using Fisher Scientific diagnostic kits. Over-production of serum interleukin-1 beta (IL-1β) level was quantified using enzyme-linked immunosorbent assay (ELISA) kits according to the manufacturer’s protocols (Mouse IL-1β ELISA Kit CAT No: RAB0274, Sigma-Aldrich, Merck KGaA, Darmstadt, Germany). Plates were read at 450 nm using an automatic microplate reader (Elx 800; Biotech United States).

### 2.14 Assessment of anti-inflammatory proficiency

Organs were homogenized using 10X buffer (1 mM EDTA and 100 mM potassium phosphate buffer; pH 7.4) followed by centrifugation for 30 min at 4°C and 12,000 × g. The supernatant was separated and analyzed for the parameters mentioned below.

#### 2.14.1 Catalase activity

Catalase (CAT) activity was analyzed by following a protocol described previously (Kazmi et al., 2018). In brief, 2,500 µL of phosphate buffer (50 mM, pH 5.0), 100 µL of the supernatant, and 400 µL of H_2_O_2_ (5.9 mM) were mixed. The change in absorbance was recorded at 240 nm after 1 min. A variation of 0.01 units per min (U/min) in absorbance was read as one unit of CAT activity. The results were expressed as units per milligram protein (U/mg protein).

#### 2.14.2 Peroxidase activity

A previously demonstrated procedure was followed for estimating peroxidase (POD) activity (Majid et al., 2019). A mixture containing 2,500 µL of phosphate buffer (50 mM, pH 5.0), 300 µL of H_2_O_2_ (50 mM), 150 µL of guaiacol (20 mM), and 1,000 µL of supernatant was read at 470 nm. The change in absorbance was recorded after 1 minute. A change of 0.01 U/min in absorbance was considered as one unit of POD activity. The results were expressed in U/mg protein.

#### 2.14.3 Superoxide dismutase activity

A previously illustrated methodology was followed to estimate superoxide dismutase (SOD) activity (Nasir et al., 2020). An aliquot of 100 µL of phenazine methosulphate (186 µM) was added to 1,200 µL of pyrophosphate buffer (0.052 mM, pH 7.0). Approximately 300 μL of supernatant (obtained after centrifuging tissue homogenates at 1,500 rpm for 10 min and then at 10,000 rpm for 15 min) was added to the reaction amalgam. The reaction was started by adding 0.2 mL of NADH (780 µM) and then stopped by adding 1,000 µL of glacial acetic acid. Absorbance was recorded at 560 nm. The results were articulated in U/mg protein.

#### 2.14.4 Reduced glutathione

A previously illustrated protocol was followed to estimate the intracellular glutathione (GSH) level (Naz et al., 2019). Approximately 1,000 µL of supernatant was precipitated utilizing 100 µL of sulfosalicylic acid (4%). The mixture was then centrifuged for 20 min at 1,200 × g and 4°C. Later, a mixture comprising 100 µL of filtrate, 2,700 µL of phosphate buffer (0.1 M, pH 7.4), and 200 µL of DTNB (100 mM) was read at 240 nm. The GSH content was determined as nanomole GSH per milligram protein (nM GSH/mg protein).

#### 2.14.5 Lipid peroxidation/thiobarbituric acid reactive substance assay

A previously illustrated protocol was followed for thiobarbituric acid reactive substances (TBARS) estimation (Khan et al., 2012). Approximately 580 µL of phosphate buffer (0.1 M, pH 7.4), 20 µL of ferric chloride (100 mM), 200 µL of ascorbic acid (100 mM), and 200 µL of the supernatant were mixed to form the reaction mixture. It was incubated utilizing a water bath at 37°C for 60 min. An aliquot of 1,000 µL of TCA (10%) was added to stop the reaction. Afterward, 1,000 µL of TBA (0.66%) was added to the Eppendorfs, which were then retained in boiling water for 20 min, placed on an ice bath, and then centrifuged for 10 min at 2,500 x. The quantity of TBARS was calculated by reading the absorbance of the supernatant compared to a reagent blank at 535 nm. The results were expressed as nanomolar TBARS per min per milligram protein (nM TBARS/min/mg protein).

#### 2.14.6 Nitrite assay

A previously described protocol was followed to measure nitrite contents (Naz et al., 2019). An equal volume (100 μL) of tissue homogenates NaOH (0.3 M) and zinc sulfate (5%) was mixed for deproteination. After centrifugation for 15 min at 6,400 × g, approximately 30 μL of the supernatant was mixed with 2 mL of Griess reagent present in the cuvette. Absorbance was recorded at 540 nm, and the nitrite contents were determined utilizing a sodium nitrite curve.

#### 2.14.7 Comet assay

DNA damage was quantified by following the previously illustrated protocol (Kazmi et al., 2022). Slides were dipped in 1% NMPA solution and allowed to become fixed at room temperature. Organs were minced using 1 mL of cold lysing solution and mixed with LMPA (85 μL). This second layer of lysate solution was gently applied to precoated slides and covered with coverslips. After cooling for 10–12 min using ice bags, a third and final coating of LMPA was applied. After exposure to a lysing solution, the slides were frozen for 2 h. After electrophoreses, these slides were stained with 1% ethidium bromide dye and visualized under the fluorescent microscope. DNA damage was calculated using CASP 1.2.3.b software (Krzysztof Końca, CaspLab.com). Observed comet parameters include comet length (CL), head length (HL), tail length (TL), %DNA in head (%DIH), and %DNA in tail (%DIT).

#### 2.14.8 Histopathological examination

Alterations in histoarchitecture were evaluated via the paraffin-embedded hematoxylin and eosin (H&E) staining procedure (Baig et al., 2022b). Fixation of the tissues was done using 10% formalin buffer. The wet fixed tissues were desiccated by an ascended alcohol series from 70% to 100%, cleaned with xylene, infiltrated with paraffin wax embedded into the paraffin wax blocks, sectioned into 3–4 µm-thick slices using a microtome, and placed on a glass slide. Afterward, the tissue sections were deparaffinized using absolute xylene, rehydrated using absolute alcohol gradient (70%–95%) and water, washed in PBS, and stained using H&E staining. Slides were read under the light microscope at 400× and photographed using a Tucsen camera.

#### 2.14.9 Immunohistochemical analysis

IHC studies were performed using a previously described protocol (Muhammad et al., 2022). Tissue slides were deparaffinized, and an enzymatic approach was followed for antigen retrieval. Slides were then washed three times using PBS, dipped in H_2_O_2_ (3%), rewashed with PBS, and then incubated for 2 h after applying normal goat serum (5%). Later, the slides were incubated overnight with the primary antibodies for NF-κB, NLRP3, TNF-α, TRX, and Nrf-2. After incubation, slides were washed using PBS, incubated with the secondary antibody for 90 min, and again incubated with an ABC Elite kit in a humidified chamber for 60 min. After washing with PBS (0.1 M), slides were stained with DAB, dehydrated using ethanol (70%, 80%, 90%, and 100%), fixed with xylene, and then covered with mounting media. Images were obtained using a light microscope and analyzed using ImageJ software 1.48 version (NIH) (Java 1.8.9_66).

### 2.15 Statistical analysis

All data obtained in this study are reported as mean ± SD. Statistical analysis software included Statistix 8.1 (one-way analysis of variance with *post hoc* Tukey’s multiple comparison test) and Origin 8.5 (graphical representation). Statistical significance was set at *p* < 0.05.

## 3 Results

### 3.1 Effects on body weights of experimental animals

The effects of artemisinic compounds on the body weights of experimental animals are illustrated in Table 1. The disease control exhibited a body weight of 22.72 ± 1.9 g and 18.78 ± 1.00 g on the second and fourth week, respectively. Artemisinic compounds attenuated this reduction in a dose-dependent manner. Among all the test moieties, maximum restorative effects compared to the positive control were produced by a high dose (HD) of ART (ARTHD); the animals weighed 29.25 ± 1.84 g in the fourth week. LDs also exhibited significant ameliorative effects compared to the disease control.

**TABLE 1:** Effects of treatments on the body weight of experimental animals.

Group	Initial body weight (g)	Body weight on day 14 (g)	Final body weight (g)
Normal control	25.12 ± 0.74^a^	27.29 ± 1.3^a^	29.12 ± 0.9^a^
Vehicle control	25.24 ± 0.62^a^	27.01 ± 0.6^a^	29.07 ± 0.7^a^
Positive control	26.24 ± 1.21^a^	22.26 ± 1^b^	28.99 ± 1.8^ab^
Disease control	26.82 ± 1.95^a^	22.72 ± 1.9^b^	18.78 ± 1^d^
ARTHD	28.01 ± 2.15^a^	22.31 ± 1^b^	29.25 ± 1.8^a^
ARTLD	27.67 ± 2.28^a^	22.81 ± 1.6^b^	26.02 ± 2.7^abc^
ARTEMHD	27.62 ± 1.94^a^	22.76 ± 1.3^b^	29.17 ± 1.9^a^
ARTEMLD	27.88 ± 1.81^a^	22.15 ± 1.4^b^	24.91 ± 1.3^bc^
ARTESHD	26.95 ± 1.45^a^	22.40 ± 0.78^b^	29.05 ± 3.3^a^
ARTESLD	27.49 ± 1.93^a^	22 ± 0.9^b^	24.43 ± 2^c^
DHAHD	27.43 ± 1.57^a^	22.43 ± 2.3^b^	29.21 ± 2.4^abc^
DHALD	27.72 ± 1.72^a^	22.42 ± 1.6^b^	25.42 ± 1.2^a^

Results are presented as mean ± SD (n = 5) Means with different superscript (a–d) letters are significantly (*p*< 0.05) different from one another, according to Tukey’s multiple comparison test. HD, high dose; LD, low dose; ART, artemisinin; ARTEM, artemether; ARTES, artesunate; DHA, dihydroartemisinin.

### 3.2 Behavioral studies

#### 3.2.1 Effects on depressive-like behavior

**FIGURE 2:**
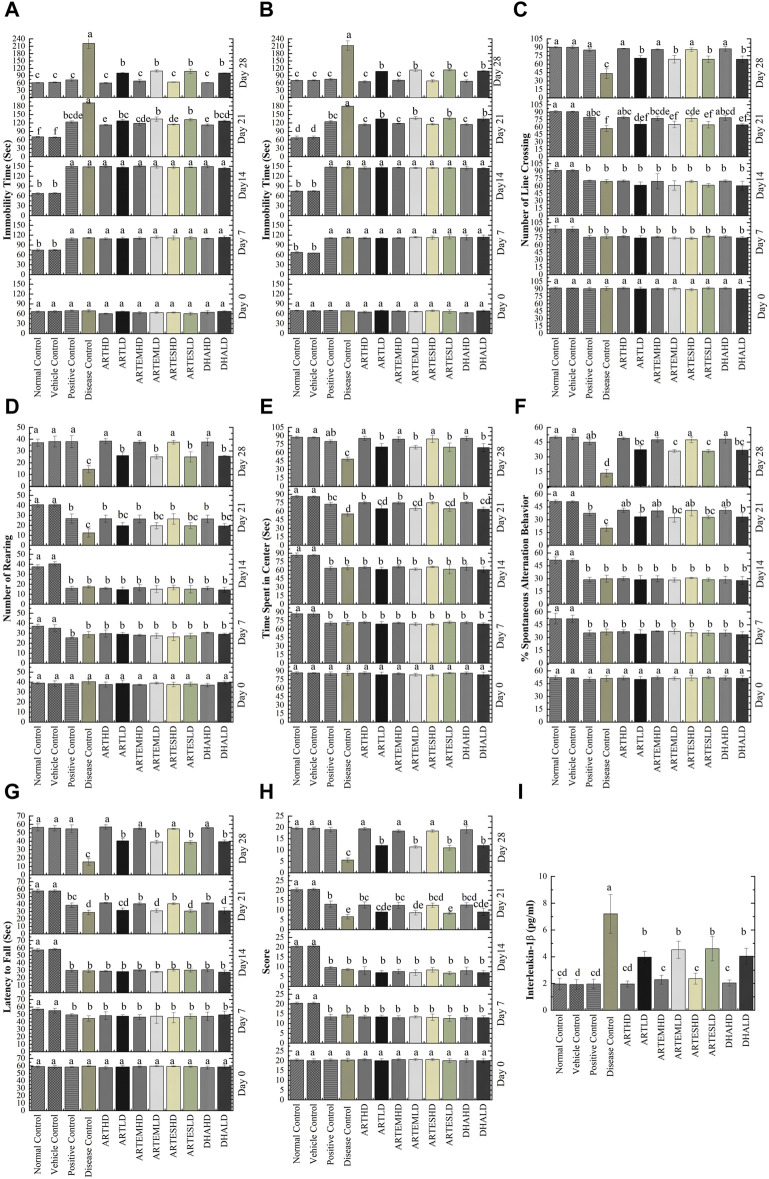
Effects of artemisinic compounds on CCl_4_-induced depression evaluated through FST **(A)** and TST **(B);** number of line crossings **(C)**, number of rearings **(D)**, and time spent in the center **(E)** evaluated through OFT; %Spontaneous alternation behavior **(F)** evaluated through Y-maze; Muscle strength **(G)** and motor activity **(H)** of experimental animals evaluated through Kondziela’s inverted screen and weightlifting test. **(I)** Effects of artemisinic compounds on CCl_4_-induced elevation of IL-1β serum levels. Data are expressed as mean ± SD (n = 5). Means with different superscript **(A–F)** letters are significantly different from one another, according to Tukey’s multiple comparison test at *p* < 0.05. HD, high dose; LD, low dose; ART, artemisinin; ARTEM, artemether; ARTES, artesunate; DHA, dihydroartemisinin.

The antidepressant potential of the ACs was evaluated through FST and TST. Increased immobility time in both studies was observed in CCl_4_-treated animals compared to the normal control on days 7 and 14 (Figures 2A, B). Treatment with artemisinic compounds significantly (*p* < 0.05) reduced immobility time in a dose-dependent manner, as observed on days 21 and 28. On day 28, the maximum reduction compared to the positive control was exhibited by ARTHD to 60.20 ± 1.30 s and 65.60 ± 3.36 s in the FST and the TST, respectively.

#### 3.2.2 Effects on anxiety-like behavior

The anxiolytic potential of artemisinic compounds was determined through the number of line crossings, number of rearings, and time spent in the center in an OFT. These parameters were found to be significantly (*p* < 0.05) reduced in CCl_4_-treated animals, who showed an increase in anxious behavior on days 7 and 14 (Figures 2C–E). Artemisinic compounds demonstrated a dose-dependent significant (*p* < 0.05) anxiolytic response compared to silymarin, with ARTHD exhibiting the maximum alleviating effects.

#### 3.2.3 Effects on exploratory behavior

The exploratory behavior of experimental animals was evaluated through the Y-maze test. Significantly (*p* < 0.05) decreased percent spontaneous alternation behavior (%SAB) was observed on days 7 and 14 in the CCl_4_-treated animals. Artemisinic compounds improved memory deficit in a dose-dependent manner, as observed on days 21 and 28 (Figure 2F). Among all the test moieties, the maximum improvement of 48.79% ± 1.19% was demonstrated by the ARTHD group compared to the positive control.

#### 3.2.4 Effects on muscle coordination and strength

The weightlifting test and Kondziela’s inverted screen test were performed to evaluate muscular strength. A significantly reduced (*p* < 0.05) latency to fall and a marked decrement in weight lifting score was observed in CCl_4_-treated animals on days 7 and 14 (Figures 2G, H, respectively). Artemisinic compounds dose-dependently alleviated the aforementioned parameters, as observed on day 28. Herein, ARTHD exhibited the maximum latency to fall and weight lifting scores of 57.00 ± 2.55 s and 19.40 ± 0.55 s, compared to the positive control.

### 3.3 Hematological profile

The effects of artemisinic compounds on CCl_4_-induced hematological deteriorations are summarized in Table 2. A decrement in Hb level (2.91 ± 0.2 g/dL), %HCT (10.67% ± 0.9%), RBCs (2.62 ± 0.2 (×106)/μL), platelet count (1.94 ± 0.2 (×105)/μL) and elevated levels of ESR (9.69 ± 0.8 mm/h) and WBCs (8.72 ± 0.7a (×103)/μL) were observed in the disease control animals in contrast to the normal control. Artemisinic compounds normalized these perturbations dose-dependently compared to the positive control. ARTHD exhibited the maximum efficiency to 11.24 ± 0.4 g/dL Hb level, 38.23% ± 1.2% HCT, 7.86 ± 0.2 (×10^6^)/µL RBCs, 6.63 ± 0.2 (×10^5^)/µL platelet count, 3.52 ± 0.1 (×10^3^)/µL WBCs and 4.42 ± 0.1 mm/h ESR. DHAHD was found to be as effective as ARTHD, followed by ARTEMHD and ARTESHD.

**TABLE 2:** Effects of treatments on the hematological parameters of experimental animals.

Group	RBCs (×10^6^)/µL	WBCs (×10^3^)/µL	Platelets (×10^5^)/µL	Hb (g/dL)	%HCT	ESR (mm/h)
Normal control	8.07 ± 0.8^a^	3.55 ± 0.3^c^	6.92 ± 0.7^a^	11.53 ± 1.2^a^	42.68 ± 4.4^a^	5.04 ± 0.4^c^
Vehicle control	8.09 ± 0.4^a^	3.61 ± 0.2^c^	6.93 ± 0.32^a^	11.56 ± 0.5^a^	42.76 ± 1.9^a^	5.08 ± 0.2^c^
Positive control	7.35 ± 0.2^a^	3.71 ± 0.1^c^	6.29 ± 0.1^a^	10.50 ± 0.2^a^	36.76 ± 0.8^b^	4.20 ± 0.1^d^
Disease control	2.62 ± 0.2^c^	8.72 ± 0.7^a^	1.94 ± 0.2^c^	2.91 ± 0.2^c^	10.67 ± 0.9^d^	9.69 ± 0.8^a^
ARTHD	7.86 ± 0.2^a^	3.52 ± 0.1^c^	6.63 ± 0.2^a^	11.24 ± 0.4^a^	38.23 ± 1.2^ab^	4.42 ± 0.1^cd^
ARTLD	5.19 ± 0.2^b^	6.12 ± 0.22^b^	4.28 ± 0.2^b^	7.04 ± 0.3^b^	24.71 ± 1^c^	7.11 ± 0.3^b^
ARTEMHD	7.59 ± 0.9^a^	3.92 ± 0.1^c^	6.38 ± 0.7^a^	10.69 ± 1.2^a^	35.95 ± 4.1^b^	4.53 ± 0.1^cd^
ARTEMLD	5.12 ± 0.6^b^	6.30 ± 0.26^b^	4.16 ± 0.5^b^	6.87 ± 0.8^b^	22.67 ± 2.6^c^	7.16 ± 0.3^b^
ARTESHD	7.47 ± 0.1^a^	4.04 ± 0.1^c^	6.29 ± 0.1^a^	10.38 ± 0.2^a^	34.87 ± 0.7^b^	4.75 ± 0.1^cd^
ARTESLD	5.03 ± 0.5^b^	6.39 ± 0.3^b^	4.12 ± 0.4^b^	6.74 ± 0.7^b^	21.89 ± 2.4^c^	7.38 ± 0.3^b^
DHAHD	7.78 ± 0.1^a^	3.75 ± 0.04^c^	6.52 ± 0.1^a^	10.92 ± 0.1^a^	37.89 ± 0.5^ab^	4.45 ± 0.1^cd^
DHALD	5.01 ± 0.8^b^	6.23 ± 0.08^b^	4.19 ± 0.7^b^	6.98 ± 1.2^b^	23.78 ± 4^c^	7.14 ± 0.1^b^

Results are presented as mean ± SD (n = 5). Means with different superscript (a–d) letters are significantly (*p*< 0.05) different from one another, according to Tukey’s multiple comparison test. RBCs, red blood cell count; WBCs, white blood cell count; Hb, hemoglobin; HCT, hematocrit; HD, high dose; LD, low dose; ART, artemisinin; ARTEM, artemether; ARTES, artesunate; DHA, dihydroartemisinin.

### 3.4 Effects on serum interleukin-1 beta levels

The discernible serum level of IL-1β observed in the disease control, 7.2 ± 1.4 pg/mL, signifies the presence of inflammation (Figure 2I). Treatment with artemisinic compounds reversed these perturbations, with ARTHD maximally restoring the IL-1β levels by 1.96 ± 0.2 pg/mL compared to the disease control.

### 3.5 Neuroprotective effects of artemisinic compounds

#### 3.5.1 Effects on endogenous antioxidant status

**FIGURE 3:**
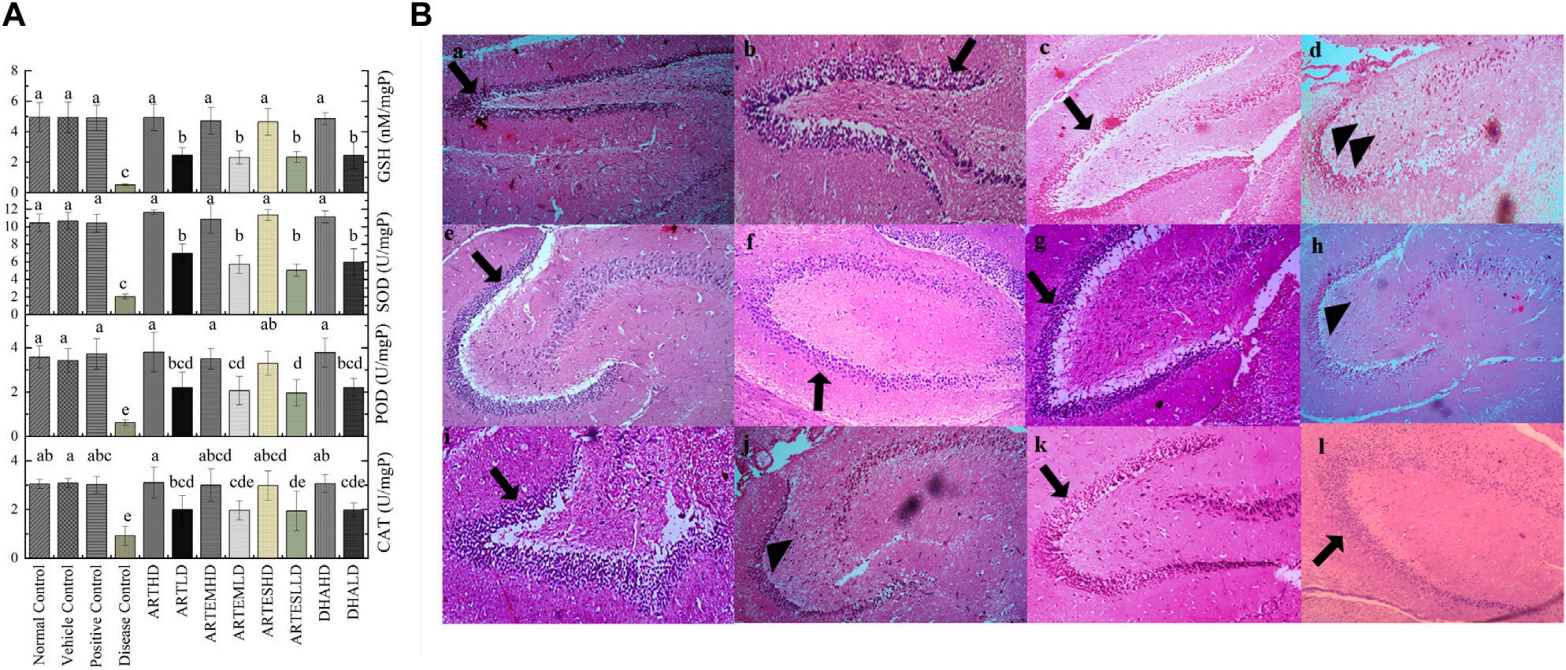
**(A)** Effects of artemisinic compounds on endogenous antioxidant levels in brain tissues. The results are presented as mean ± SD (n = 5). Means with different superscript (a–e) letters are significantly different from one another, according to Tukey’s multiple comparison test at *p* < 0.05. **(B)** Hematoxylin and eosin-stained brain tissue: (a) normal control, (b) vehicle control, (c) positive control, (d) disease control, (e) ARTHD, (f) ARTLD, (g) ARTEMHD, (h) ARTEMLD, (i) ARTESHD, (j) ARTESLD, (k) DHAHD, and (l) DHALD. The arrowheads point to the thinner granule cell layer of the dentate gyrus, whereas the arrows point to the normal granule cell layer of the dentate gyrus. HD, high dose; LD, low dose; ART, artemisinin; ARTEM, artemether; ARTES, artesunate; DHA, dihydroartemisinin.

Almost-slashed endogenous antioxidant levels were observed in brain tissues of the disease control as compared to the normal control: 0.92 ± 0.4 U/mg, 0.62 ± 0.13 U/mg, and 2.03 ± 0.27 U/mg protein activity levels of CAT, POD, and SOD, respectively. GSH content was also significantly (p < 0.05) reduced to 0.51 ± 0.05 nM/mg protein compared to the normal control (Figure 3A). Artemisinic compounds dose-dependently escalated the activities of endogenous antioxidants, with ARTHD exhibiting the maximum restoration of CAT, POD, and SOD to 3.1 ± 0.64 U/mg, 3.8 ± 0.9 U/mg, and 11.65 ± 0.25 U/mg protein, respectively. The GSH level was also maximally restored by ARTHD to 4.93 ± 0.9 nM/mg protein.

#### 3.5.2 Expression of nitrite and TBARS levels

The effects of artemisinic compounds on nitrite and TBARS levels in brain homogenates are summarized in Table 3. The highest nitrite (87.47 ± 11.37 μM) and TBARS (133.99 ± 17.3 nM/min/mg protein) content were exhibited by the disease control group. Artemisinic compounds significantly (*p* < 0.05) decreased this hike in a dose-dependent manner. Herein, ARTHD maximally reduced nitrite and TBARS levels by 40.27 ± 2.8 μM and 20.23 ± 1.6 nM/min/mg protein, respectively, compared to silymarin.

**TABLE 3:** Effects of treatments on the expression of TBARS and nitrite levels in the experimental animals.

Group	TBARS (nM/min/mg protein)			Nitrite (μM)				
	Brain	Heart	Liver	Kidney	Brain	Heart	Liver	Kidney
Normal control	21.56 ± 2.4^c^	17.32 ± 1.5^c^	24.63 ± 1.1^c^	14.62 ± 2.8^c^	39.73 ± 5.6^c^	40.93 ± 4.02^c^	52.27 ± 7.3^c^	46.8 ± 6.6^c^
Vehicle control	21.65 ± 3.1^c^	17.32 ± 1.9^c^	24.53 ± 3.5^c^	14.80 ± 3.3^c^	39.47 ± 5.2^c^	37.87 ± 4.9^c^	51.87 ± 6.9^c^	46.67 ± 6.1^c^
Positive control	22.45 ± 3.6^c^	17.66 ± 2.9^c^	25.24 ± 1.9^c^	15.35 ± 1.9^c^	40.53 ± 5.4^c^	37.6 ± 4.7^c^	51.6 ± 6.6^c^	46.4 ± 5.9^c^
Disease control	134 ± 17.3^a^	104 ± 6.5^a^	161.9 ± 17^a^	105.6 ± 15^a^	87.47 ± 11.3^a^	102.53 ± 17.3^a^	135.33 ± 5.7^a^	128.93 ± 8.3^a^
ARTHD	20.23 ± 1.6^c^	15.52 ± 1.9^c^	22.79 ± 3.0^c^	13.07 ± 2.7^c^	40.27 ± 2.8^c^	39.73 ± 2.9^c^	51.87 ± 3.4^c^	45.07 ± 2.2^c^
ARTLD	42.72 ± 4.7^b^	33.45 ± 1.8^b^	52.51 ± 4.3^b^	25.66 ± 3.2^b^	63.07 ± 5.7^b^	71.06 ± 5.0^b^	90.93 ± 5.2^b^	86.8 ± 4.6^b^
ARTEMHD	22.3 ± 3.13^c^	17.61 ± 7.7^c^	25.47 ± 2.5^c^	14.76 ± 2.2^c^	44 ± 5.8^c^	40.8 ± 5.9^c^	53.47 ± 4.7^c^	48.13 ± 5.6^c^
ARTEMLD	46.78 ± 2.3^b^	37.50 ± 5.5^b^	53.29 ± 2.2^c^	26.05 ± 2.5^b^	66.8 ± 5.1^b^	72.13 ± 5.3^b^	93.6 ± 7.87^b^	88.8 ± 6.7^b^
ARTESHD	24.88 ± 1.8^c^	18.67 ± 0.7^c^	28.6 ± 2.6^c^	15.21 ± 1.9^c^	45.47 ± 5.9^c^	42.13 ± 2.7^c^	54.13 ± 4.2^c^	49.07 ± 1.9^c^
ARTESLD	47.16 ± 6.6^b^	40.10 ± 2.2^b^	57.47 ± 1.9^b^	27.98 ± 0.2^b^	67.33 ± 6.5^b^	74.13 ± 4.8^b^	94.93 ± 2.7^b^	90.27 ± 6.4^b^
DHAHD	21.16 ± 2.9^c^	16.10 ± 2.6^c^	24.01 ± 1.9^c^	13.64 ± 1.6^c^	41.33 ± 2.9^c^	40.53 ± 1.4^c^	52.8 ± 3.1^c^	46.67 ± 4.9^c^
DHALD	45.74 ± 2.5^b^	33.89 ± 1.4^b^	53.61 ± 2.4^b^	25.98 ± 1.1^b^	63.07 ± 2.7^b^	72.13 ± 3.9^b^	91.07 ± 3.9^b^	87.07 ± 5.2^b^

Results are presented as mean ± SD (n = 5). Means with different superscript (a–c) letters are significantly (*p*< 0.05) different from one another, according to Tukey’s multiple comparison test. TBARS, thiobarbituric acid reactive substances; HD, high dose; LD, low dose; ART, artemisinin; ARTEM, artemether; ARTES, artesunate; DHA, dihydroartemisinin.

#### 3.5.3 Effects on brain histology

Histological analysis of brain sections is illustrated in Figure 3B. The normal control exhibited a smooth architecture with normal dentate gyrus, neuronal, and glial cells. The disease control group exhibited pyknotic nuclei, vacuolation, swollen cells, and a marked decrease in the cellularity of the granular layer. Artemisinic compounds dose-dependently relieved the CCl_4_-induced neuroinflammation compared to silymarin. A normal picture with a denser granular layer comprising closely packed neurocytes with well-defined, rounded vesicular nuclei was revealed by groups treated with HDs compared to the disease control. LD-treated groups also eliminated the toxicological effects depicting mild degeneration of the neurons, fewer pyknotic nuclei, and a comparatively dense granular layer.

### 3.6 Cardioprotective effects of artemisinic compounds

#### 3.6.1 Effects on serological parameters

Serum levels of CK and LDH were evaluated to analyze the cardioprotective effects of artemisinic compounds (Table 4). The disease control demonstrated a significant (*p* < 0.05) increase in both serum CK (789.38 ± 10.9 U/l) and LDH (925.76 ± 12.85 U/l) levels compared to the normal control. Artemisinic compounds dose-dependently decreased this elevation, with ARTHD exhibiting the maximal abatement of CK (87.54 ± 2.15 U/l) and LDH (321.96 ± 7.9 U/l).

**TABLE 4:** Effects of treatment on the serological parameters of experimental animals.

Group	ALT (U/l)	AST (U/l)	Albumin (g/dL)	Urea (mg/dL)	Creatinine (mg/dL)	LDH (U/l)	CK (U/l)
Normal control	43.01 ± 0.7^fg^	77.02 ± 1.7^c^	4.61 ± 0.5^a^	44.01 ± 0.9^c^	0.50 ± 0.01^c^	298.11 ± 6.44^ef^	82.03 ± 1.8^c^
Vehicle control	42.23 ± 1.9^g^	78.03 ± 1.7^c^	4.62 ± 0.2^a^	44.02 ± 0.7^c^	0.50 ± 0.01^c^	293.18 ± 4.6^f^	82.04 ± 1.3^c^
Positive control	47.03 ± 1.4^ef^	82.03 ± 1.8^c^	4.20 ± 0.2^a^	47.01 ± 1^c^	0.54 ± 0.01^c^	327.12 ± 7.1^cde^	89.03 ± 1.9^c^
Disease control	147.03 ± 2.3^a^	215.34 ± 17.5^a^	0.77 ± 0.1^c^	91.91 ± 17.1^a^	3.2 ± 0.6^a^	925.76 ± 12.9^a^	789.38 ± 10.9^a^
ARTHD	45.39 ± 1.3^efg^	80.49 ± 1.9^c^	4.49 ± 0.1^a^	46.28 ± 1.1^c^	0.52 ± 0.01^c^	321.96 ± 7.9^def^	87.54 ± 2.15^c^
ARTLD	95.48 ± 2.5^c^	150.04 ± 5.3^b^	2.6 ± 0.1^b^	69.77 ± 2.5^b^	1.83 ± 0.07^b^	630.76 ± 22.4^b^	437.71 ± 15.6^b^
ARTEMHD	48.86 ± 0.9^de^	86.56 ± 2^c^	4.27 ± 0.5^a^	49.32 ± 1.1^c^	0.56 ± 0.01^c^	348.24 ± 8.1^cd^	95.61 ± 2.2^c^
ARTEMLD	98.68 ± 3.4^bc^	153.89 ± 4.9^b^	2.52 ± 0.3^b^	71.62 ± 2.9^b^	1.91 ± 0.08^b^	647.40 ± 26.5^b^	444.79 ± 18.2^b^
ARTESHD	52.00 ± 0.5^d^	88.94 ± 1.2^c^	4.15 ± 0.1^a^	50.53 ± 1^c^	0.59 ± 0.01^c^	356.76 ± 4.8^c^	100.05 ± 1.4^c^
ARTESLD	101.02 ± 1.3^b^	157.49 ± 3.6^b^	2.47 ± 0.3^b^	72.15 ± 3.2^b^	1.98 ± 0.09^b^	655.92 ± 29.9^b^	450.95 ± 20.6^b^
DHAHD	46.23 ± 1.2^efg^	82.04 ± 0.9^c^	4.46 ± 0.1^a^	48.62 ± 0.6^c^	0.53 ± 0.01^c^	334.25 ± 3.9^cd^	90.15 ± 1^c^
DHALD	97.83 ± 2.5^bc^	150.52 ± 1.6^b^	2.58 ± 0.4^b^	70.5 ± 0.7^b^	1.88 ± 0.02^b^	640.34 ± 7.9^b^	442.58 ± 5.4^bc^

Results are presented as mean ± SD (n = 5). Means with different superscript (a–g) letters are significantly (*p*< 0.05) different from one another, according to Tukey’s multiple comparison test. ALT, alanine transaminase; AST, aspartate transferase; LDH, lactate dehydrogenase; CK, creatine kinase; HD, high dose; LD, low dose; ART, artemisinin; ARTEM, artemether; ARTES, artesunate; DHA, dihydroartemisinin.

#### 3.6.2 Effects on endogenous antioxidants

**FIGURE 4:**
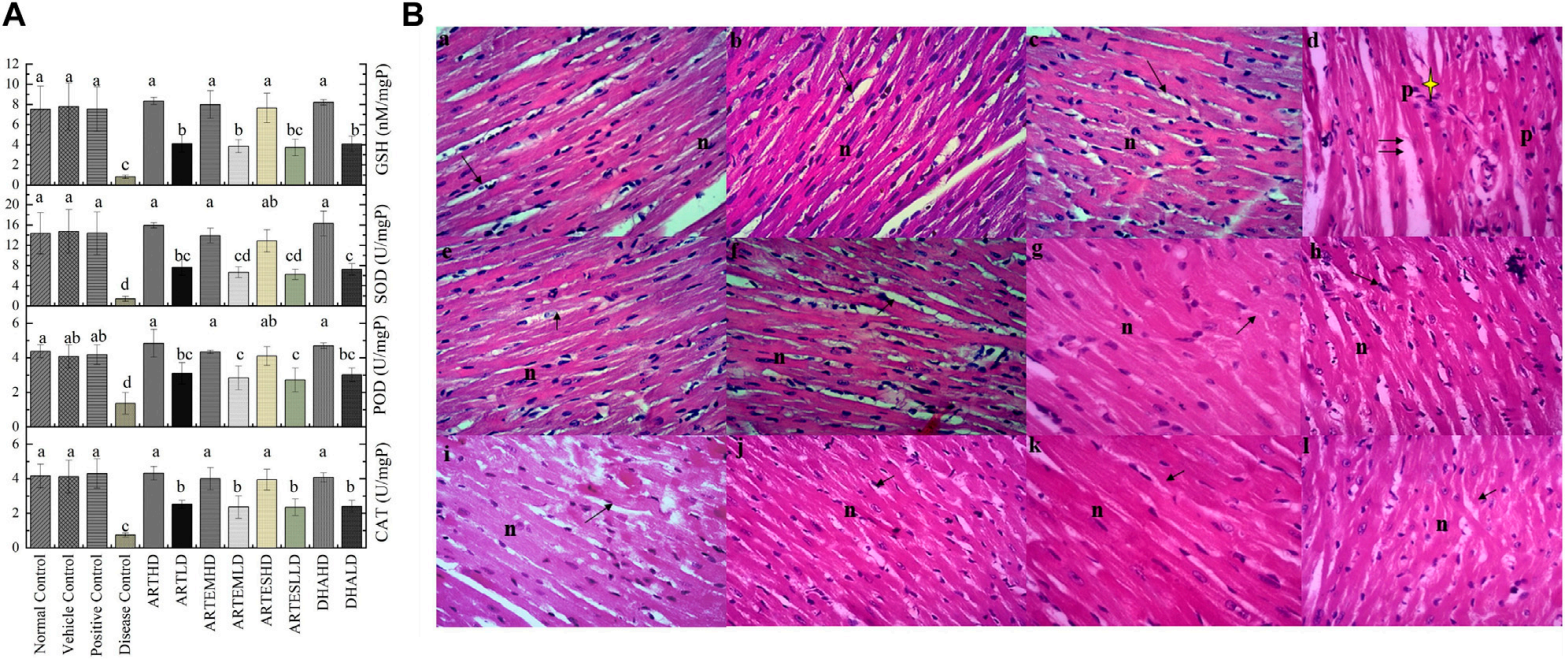
**(A)** Effects of artemisinic compounds on endogenous antioxidant levels in cardiac tissues. The results are presented as mean ± SD (n = 5). Means with different superscript (a–d) letters are significantly different from one another, according to Tukey’s multiple comparison test at *p* < 0.05. **(B)** Hematoxylin and eosin (H&E) stained cardiac cross sections: (a) normal control, (b) vehicle control, (c) positive control, (d) disease control, (e) ARTHD, (f) ARTLD, (g) ARTEMHD, (h) ARTEMLD, (i) ARTESHD, (j) ARTESLD, (k) DHAHD, and (l) DHALD. The double arrows point to increased spacing between cells, n shows central vesicular nuclei, the single arrows point to striated eosinophilic sarcoplasm, and the yellow star identifies periventricular infiltration of inflammatory cells. HD, high dose; LD, low dose; ART, artemisinin; ARTEM, artemether; ARTES, artesunate; DHA, dihydroartemisinin.

The effects of artemisinic compounds on cardiac enzymatic levels are presented in Figure 4A. Compared to the normal control, the disease control significantly (*p* < 0.05) decreased the activity levels of CAT, POD, and SOD to 0.74 ± 0.11 U/mg, 1.36 ± 0.62 U/mg, and 1.41 ± 0.5 U/mg protein, respectively. Depleted GSH content was also observed in the disease control at 0.81 ± 0.2 nM/mg protein. The AC-treated groups exhibited a significant (*p* < 0.05) and dose-dependent rise in antioxidant levels with the HD of ART maximally attenuating the activity levels of CAT, POD, and SOD to 4.32 ± 0.39 U/mg, 4.84 ± 0.8 U/mg, and 15.91 ± 0.5 U/mg protein. Additionally, the GSH content was also restored to 8.34 ± 0.4 nM/mg protein in the ARTHD-treated group. The vehicle control did not exhibit any toxicity.

#### 3.6.3 Expression of nitrite and TBARS levels

The nitrite and TBARS contents of cardiac tissues are illustrated in Table 3. A significant (*p* < 0.05) escalation in myocardial nitrite and TBARS levels was observed in the disease control to 102.53 ± 17.3 μM and 104 ± 6.5 nM/min/mg protein, respectively, compared to the normal control. Artemisinic compounds produced inhibitory effects in a dose-dependent manner, with ARTHD exhibiting the maximum restoration to 39.73 ± 2.9 μM of nitrite and 15.52 ± 1.9 nM/min/mg protein of TBARS.

#### 3.6.4 Effects on cardiac histoarchitecture

The cardiac histology (Figure 4B) of the normal control exhibited a branched and striated myofibrillar structure. The disease control exhibited severe cardiotoxicity with significant pathological changes, including inflammation, vacuolization, eosinophilic infiltration in the cytoplasm, hyaline necrosis, and loss of proper striations compared to the normal control. Artemisinic compounds neutralized the CCl_4_-induced distortion. Herein, cardiac sections from the groups treated with HDs exhibited normal histoarchitecture with branched and striated myofibrils. Mild vacuolization can be observed in LD-treated groups.

### 3.7 Hepatoprotective effects of artemisinic compounds

#### 3.7.1 Effects on serological parameters

Serum levels of the hepatic inflammatory markers ALT and AST were also assessed (Table 4). A marked elevation was observed in the disease control compared to the normal control to 147.03 ± 2.3 U/l and 215.34 ± 17.5 U/l of ALT and AST, respectively. Artemisinic compounds dose-dependently reduced the elevated levels of hepatic stress biomarkers, with ARTHD exhibiting the maximum effects to 45.39 ± 1.3 U/l and 80.49 ± 1.9 U/l of ALT and AST, respectively.

#### 3.7.2 Effects on endogenous antioxidants

**FIGURE 5:**
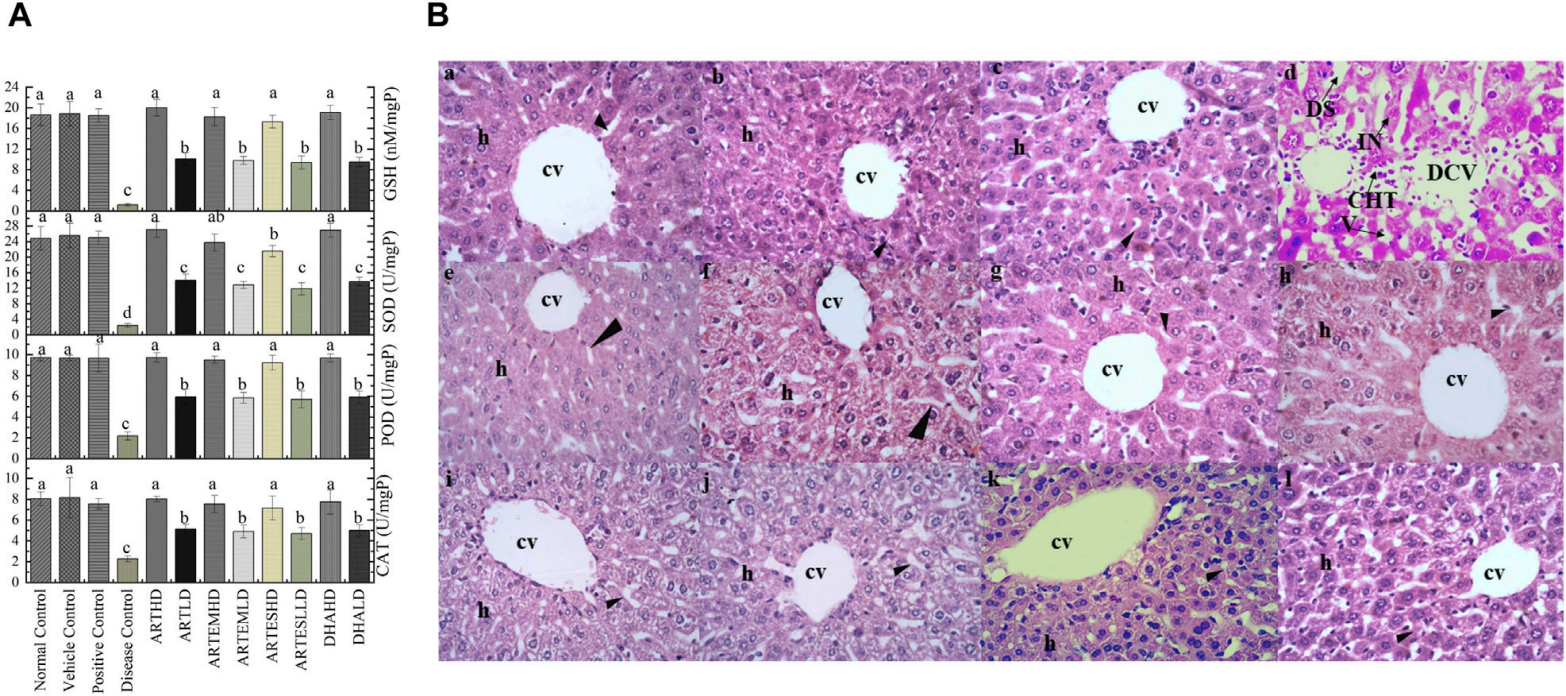
**(A)** Effects of artemisinic compounds on endogenous antioxidant levels in hepatic tissues. The results are presented as mean ± SD (n = 5). Means with different superscript (a–d) letters are significantly different from one another, according to Tukey’s multiple comparison test at *p* < 0.05. **(B)** Hematoxylin and eosin-stained hepatic cross sections: (a) normal control, (b) vehicle control, (c) positive control, (d) disease control, (e) ARTHD, (f) ARTLD, (g) ARTEMHD, (h) ARTEMLD, (i) ARTESHD, (j) ARTESLD, (k) DHAHD, and (l) DHALD. cv, central vein; h, hepatocytes; DCV, damaged central vein; IN, inflammation; DS, dilated sinusoids; CHT, cellular hypertrophy; HD, high dose; LD, low dose; ART, artemisinin; ARTEM, artemether; ARTES, artesunate; DHA, dihydroartemisinin.

Hepatic tissue homogenates of the disease control exhibited a marked reduction in the activity levels of CAT, POD, and SOD compared to the normal control to 2.26 ± 0.29 U/mg, 2.18 ± 0.37 U/mg, and 2.40 ± 0.5 U/mg protein, respectively. Reduced GSH level was also observed in the disease control at 1.18 ± 0.2 nM/mg protein (Figure 5A). Artemisinic compounds restored these diminished enzymatic levels in a dose-dependent manner. Herein, ARTHD exhibited maximum ameliorating effects compared to the positive control to 8.02 ± 0.2 U/mg, 9.72 ± 0.45 U/mg, and 27.11 ± 2.0 U/mg protein of CAT, POD, and SOD and 20.00 ± 0.32 nM/mg protein of GSH.

#### 3.7.3 Expression of nitrite and TBARS levels

The highest nitrite and TBARS levels were found in the liver homogenates of the disease control at 135.33 ± 5.7 μM and 161.9 ± 17 nM/min/mg protein, respectively (Table 3). Herein, both the HD and the LD groups of all the ACs demonstrated significant abrogating effects. The maximum restoration of nitrite and TBARS levels was depicted by ARTHD to 51.87 ± 3.4 μM and 22.79 ± 3 nM/min/mg protein, respectively.

#### 3.7.4 Effects on hepatic histoarchitecture

Hepatic histological analysis confirmed the restorative effects of artemisinic compounds in CCl_4_-induced hepatic damage (Figure 5B). Intense hepatic hypertrophy, cytoplasmic inflammation, vacuolization, centrilobular necrosis, and a distorted portal vein were observed in the disease control. The liver sections of groups treated with HDs of ACs revealed smooth histoarchitecture with a normal central vein, hepatocytes, and sinusoids, whereas liver sections of the LD groups revealed the presence of mild hepatic necrosis induced by CCl_4_. The normal histoarchitecture of the vehicle control portrayed its safe and non-toxic behavior.

### 3.8 Nephroprotective effects of artemisinic compounds

#### 3.8.1 Effects on serological parameters

Increased levels of urea and creatinine and reduced albumin levels were observed in the disease control at 91.91 ± 17.1, 3.2 ± 0.6 mg/dL, and 0.77 ± 0.1 g/dL, respectively (Table 4). Artemisinic compounds dose-dependently controlled these levels, with ARTHD exhibiting the maximum restoration to 46.28 ± 1.1 mg/dL of urea, 0.52 ± 0.01 mg/dL of creatinine, and 4.49 ± 0.1 g/dL of albumin. Herein, ARTHD was followed by DHAHD, ARTEMHD, and ARTESHD in approximately all the parameters tested.

#### 3.8.2 Effects on endogenous antioxidants

**FIGURE 6:**
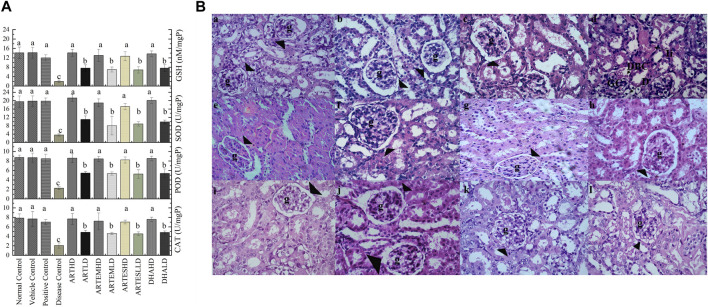
**(A)** Effects of artemisinic compounds on endogenous antioxidant levels in the kidney tissues. The results are presented as mean ± SD (n = 5). Means with different superscript (a–c) letters are significantly different from one another, according to Tukey’s multiple comparison test at *p* < 0.05. **(B)** Hematoxylin and eosin (H&E) stained kidney cross sections: (a) normal control, (b) vehicle control, (c) positive control, (d) disease control, (e) ARTHD, (f) ARTLD, (g) ARTEMHD, (h) ARTEMLD, (i) ARTESHD, (j) ARTESLD, (k) DHAHD, and (l) DHALD. The arrowheads point to Bowman’s capsules, BCD identifies Bowman’s capsule’s disruption, D identifies desquamation, g identifies glomerulus, GC identifies glomerulus congestion, and Ii identifies interstitial inflammation. HD, high dose; LD, low dose; ART, artemisinin; ARTEM, artemether; ARTES, artesunate; DHA, dihydroartemisinin.

Compared to the normal control, the disease control depicted decreased renal endogenous antioxidant activity levels to 2.06 ± 0.6 U/mg, 2.26 ± 0.3 U/mg, and 3.55 ± 0.6 U/mg protein of CAT, POD, and SOD, respectively. An almost-depleted GSH level was also observed in the disease control at 1.95 ± 0.5 nM/mg protein (Figure 6A). Dose-graded treatment with artemisinic compounds attenuated these levels compared to the disease control. Herein, ARTHD exhibited the maximum alleviation of CAT, POD, and SOD activity levels to 7.68 ± 1.2 U/mg, 8.6 ± 0.9 U/mg, and 21.29 ± 1.7 U/mg protein, respectively. GSH content was also revitalized following ARTHD treatment to 14.12 ± 1.5 nM/mg protein.

#### 3.8.3 Expression of nitrite and TBARS levels

Significantly (*p* < 0.05) increased nitrite (128.93 ± 8.3 μM) and TBARS (105.6 ± 15 nM/min/mg protein) levels were observed in the disease control compared to the normal control (Table 3). Herein, ARTHD exhibited maximum amelioration to 45.07 ± 2.2 μM and 13.07 ± 2.7 nM/min/mg protein of nitrite and TBARS, respectively. ARTEM and ARTES also exhibited restorative effects on nitrite and TBARS levels in a dose-dependent manner.

#### 3.8.4 Effects on renal histoarchitecture

The renal histology of the normal control revealed smooth Bowman’s capsules, glomerulus, and tubules (Figure 6B). The disease control demonstrated vacuolization, degeneration of tubular structures, and irregular tubular architecture with severe necrosis of epithelial cells. Groups treated with LDs demonstrated normal renal morphology with occasional tubular necrosis. Groups treated with HDs showed normal histology characterized by intact Bowman’s capsules, glomerulus, epithelial cells, and tubules.

### 3.9 Effects on DNA integrity

**FIGURE 7:**
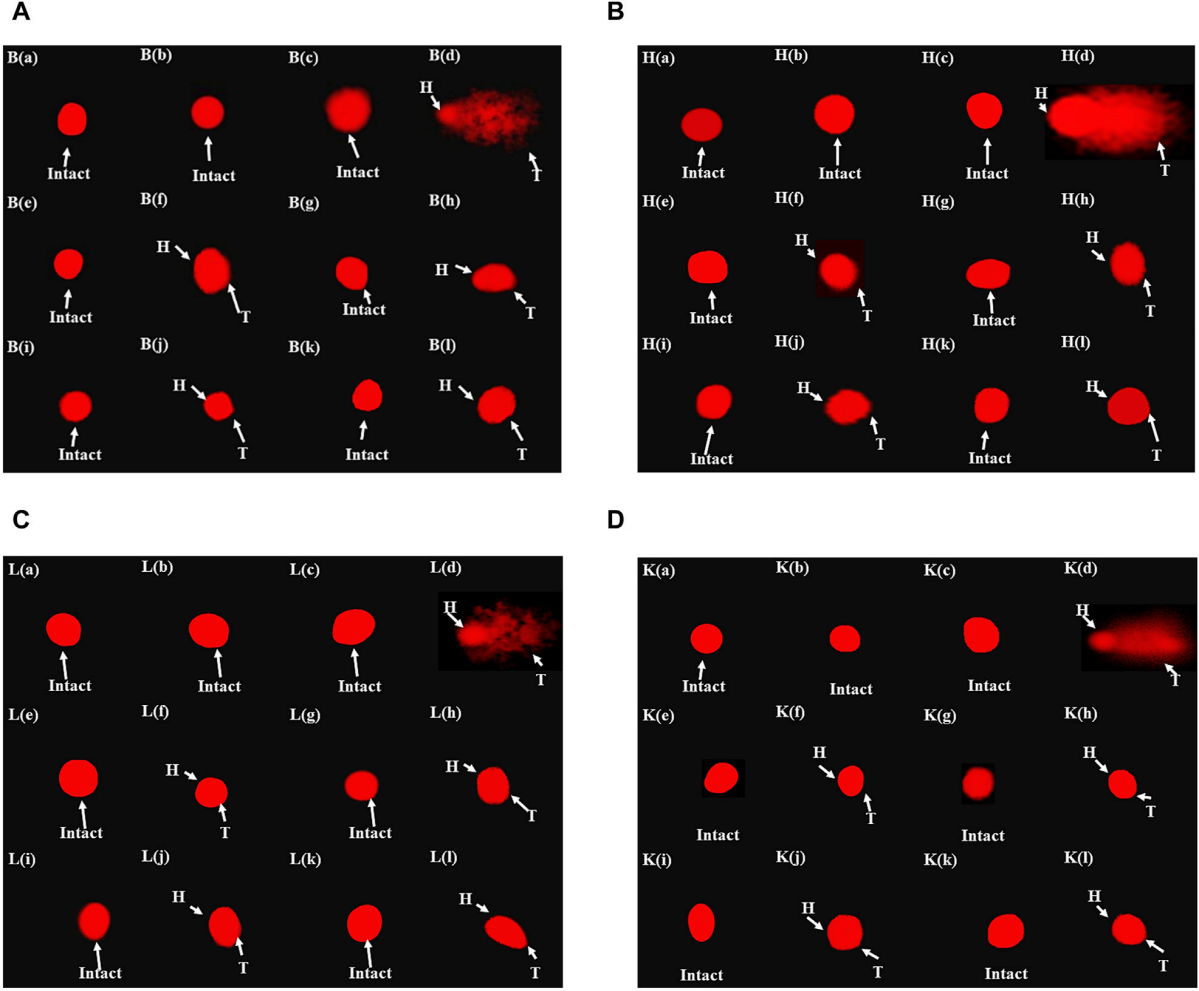
Fluorescence photomicrograph demonstrating the effects of artemisinic compounds against CCl_4_-induced DNA damage: **(A)** brain tissues, **(B)** cardiac tissues, **(C)** hepatic tissues, **(D)** kidney tissues. (a) Normal control, (b) vehicle control, (c) positive control, (d) disease control, (e) ARTHD, (f) ARTLD, (g) ARTEMHD, (h) ARTEMLD, (i) ARTESHD, (j) ARTESLD, (k) DHAHD, and (l) DHALD. HD, high dose; LD, low dose; ART, artemisinin; ARTEM, artemether; ARTES, artesunate; DHA, dihydroartemisinin.

Table 5 illustrates the genoprotective effects of artemisinic compounds. Maximum DNA damage was observed in the disease control, which is quite evident from DNA migration into the comet’s tail at rates of 52.76% ± 3.2%, 48.32% ± 3.1%, 51.85% ± 3.8%, and 50.58% ± 5.7% in brain, cardiac, hepatic, and renal tissues, respectively. Artemisinic compounds dose-dependently resisted the DNA damage compared to the disease control. The results produced by HDs of nearly all the ACs were equivalent to silymarin (Figure 7). The vehicle control did not induce any DNA toxicity.

**TABLE 5:** Genoprotective effects of artemisinic compounds.

	Comet assay												
	Sample	CL (µm)	HL (µm)	TL (µm)	%DIH	%DIT		Sample	CL (µm)	HL (µm)	TL (µm)	%DIH	%DIT
Brain	NC	52 ± 1^cd^	48 ± 1^bc^	4 ± 0.7^de^	92.31 ± 1.3^ab^	7.69 ± 1.3^de^	Liver	NC	52.8 ± 0.8^de^	49 ± 1^ab^	3.8 ± 0.4^c^	92.8 ± 0.9^a^	7.19 ± 0.9^c^
	VC	51 ± 1^d^	47 ± 1^cde^	4 ± 0.7^de^	92.16 ± 1.3^b^	7.84 ± 1.3^de^		VC	52.2 ± 0.8^e^	48.1 ± 0.5^ab^	3.8 ± 0.8^c^	92.74 ± 1.5^a^	7.27 ± 1.5^c^
	PC	52 ± 2^cd^	46 ± 1^cde^	6 ± 1.4^cd^	88.83 ± 1.9^bc^	11.16 ± 1.9^cd^		PC	54.8 ± 1.6^bcd^	50 ± 1^a^	4.8 ± 1.1^c^	91.27 ± 1.8^a^	8.73 ± 1.8^c^
	DC	66 ± 1^a^	31.2 ± 2.5^f^	34.8 ± 1.8^a^	47.24 ± 3.2^e^	52.76 ± 3.2^a^		DC	66 ± 1^a^	31.8 ± 2.8^d^	34.2 ± 2.2^a^	48.15 ± 3.8^c^	51.85 ± 3.8^a^
	ARTH	53.4 ± 1.5^b^	50.4 ± 1.1^ab^	3 ± 0.7^e^	94.39 ± 1.2^a^	5.6 ± 1.2^e^		ARTH	53.4 ± 1.5^cde^	49.2 ± 0.8^ab^	4.2 ± 1.1^c^	92.17 ± 1.9^a^	7.83 ± 1.9^c^
	ARTL	53.6 ± 1.8^bc^	45 ± 0.7^de^	8.6 ± 1.3^bc^	84 ± 2^cd^	15.99 ± 2^bc^		ARTL	55.4 ± 1.7^bc^	44.6 ± 0.5^c^	10.8 ± 1.5^b^	80.55 ± 2.1^b^	19.45 ± 2.1^b^
	ARTEMH	55.6 ± 1.3^b^	51.4 ± 1.5^a^	4.2 ± 1.3^de^	91.46 ± 2.3^ab^	7.54 ± 2.3^de^		ARTEMH	51.4 ± 0.9^e^	47 ± 0.7^b^	4.4 ± 0.5^c^	91.45 ± 0.9^a^	8.55 ± 0.9^c^
	ARTEML	55.4 ± 2.3^b^	46 ± 1.7^cde^	9.4 ± 3.4^b^	83.18 ± 5.5^d^	16.81 ± 5.5^b^		ARTEML	55.6 ± 0.5^b^	43.4 ± 1.5^c^	12.2 ± 1.3^b^	78.05 ± 2.4^b^	21.95 ± 2.4^b^
	ARTESH	52 ± 1^cd^	47.4 ± 0.9^cd^	4.6 ± 0.5^de^	91.15 ± 1^ab^	8.84 ± 1^de^		ARTESH	52.6 ± 0.5^de^	47.4 ± 0.5^b^	5.2 ± 0.4^c^	90.12 ± 0.8^a^	9.88 ± 0.8^c^
	ARTESL	55.8 ± 0.8^b^	45.4 ± 0.9^cde^	10.4 ± 1.1^b^	81.37 ± 1.9^d^	18.62 ± 1.9^b^		ARTESL	55.4 ± 0.9^bc^	43 ± 1.4^c^	12.4 ± 1.3^b^	77.62 ± 2.4^b^	22.38 ± 2.4^b^
	DHAH	54.4 ± 1.5^bc^	50.6 ± 0.5^a^	3.8 ± 1^de^	93.05 ± 1.8^a^	6.95 ± 1.8^e^		DHAH	52.6 ± 0.5^de^	48.8 ± 0.4^ab^	3.8 ± 0.4^c^	92.78 ± 0.8^a^	7.22 ± 0.8^c^
	DHAL	53.4 ± 1.7^bcd^	44.6 ± 0.5^e^	8.8 ± 1.8^bc^	83.59 ± 2.9^cd^	16.41 ± 2.9^bc^		DHAL	55.2 ± 1.6^bc^	44.2 ± 0.4^c^	11 ± 1.2^b^	80.11 ± 1.6^b^	19.89 ± 1.6^b^
Heart	NC	54.6 ± 1.1^bcde^	50.6 ± 1.8^a^	4 ± 2.3^c^	92.72 ± 4.1^a^	7.28 ± 4.1^c^	Kidney	NC	53.4 ± 1.1^cd^	50.6 ± 1.1^ab^	2.8 ± 0.4^c^	94.76 ± 0.8^a^	5.24 ± 0.8^c^
	VC	53.6 ± 1.1^cdef^	50 ± 1.4^ab^	3.6 ± 1.8^c^	93.32 ± 3.3^a^	6.68 ± 3.3^c^		VC	52.4 ± 1.1^d^	50 ± 1^ab^	2.4 ± 1.3^c^	95.45 ± 2.5^a^	4.55 ± 2.5^c^
	PC	53.6 ± 1.8^cdef^	49.6 ± 1.1^ab^	4 ± 1.6^c^	92.59 ± 2.8^a^	7.41 ± 2.8^c^		PC	53.6 ± 1.8^cd^	49.4 ± 1.1^b^	4.2 ± 2.7^c^	92.28 ± 4.7^a^	7.72 ± 4.7^c^
	DC	59.6 ± 1.1^a^	30.8 ± 1.9^e^	29 ± 1.9^a^	51.68 ± 3.1^c^	48.32 ± 3.1^a^		DC	68 ± 1^a^	33.6 ± 3.8^d^	34.4 ± 3.9^a^	49.42 ± 5.7^c^	50.58 ± 5.7^a^
	ARTH	53.8 ± 1.6^cdef^	50.8 ± 1.3^a^	3 ± 1.2^c^	94.45 ± 2.2^a^	5.5 ± 2.2^c^		ARTH	55 ± 1.9^bcd^	52 ± 1.4^ab^	3 ± 0.7^c^	94.57 ± 1.2^a^	5.43 ± 1.15^c^
	ARTL	55.2 ± 1.3^bcd^	43.8 ± 0.8^d^	11.4 ± 1.1^b^	79.37 ± 1.7^b^	20.63 ± 1.7^b^		ARTL	55.2 ± 1.3^bc^	44.6 ± 1.1^c^	10.6 ± 1.5^b^	80.82 ± 2.5^b^	19.18 ± 2.5^b^
	ARTEMH	52.2 ± 1.4^ef^	48 ± 0.7^bc^	4.2 ± 1.1^c^	91.99 ± 1.9^a^	8 ± 1.9^c^		ARTEMH	57.4 ± 0.9^b^	53 ± 1^a^	4.4 ± 0.9^c^	92.34 ± 1.5^a^	7.66 ± 1.5^c^
	ARTEML	56 ± 1.9^bc^	43.4 ± 1.3^d^	12.6 ± 2.5^b^	77.58 ± 3.9^b^	22.42 ± 3.9^b^		ARTEML	57 ± 1.9^b^	45 ± 1.2^c^	12 ± 2.1^b^	79 ± 3.2^b^	20.99 ± 3.2^b^
	ARTESH	51.6 ± 0.5^f^	46.8 ± 0.8^c^	4.8 ± 0.4^c^	90.69 ± 0.9^a^	9.31 ± 0.9^c^		ARTESH	53.4 ± 0.5^cd^	49 ± 0.7^b^	4.4 ± 0.5^c^	91.76 ± 1^a^	8.24 ± 1^c^
	ARTESL	57.2 ± 0.4^ab^	43.8 ± 1.9^d^	13.4 ± 2.1^b^	76.58 ± 3.6^b^	23.42 ± 3.6^b^		ARTESL	57.2 ± 0.4^b^	44.2 ± 1.5^c^	13 ± 1.9^b^	77.29 ± 3.1^b^	22.71 ± 3.1^b^
	DHAH	52.8 ± 1.3^def^	49.4 ± 0.5^abc^	3.4 ± 0.9^c^	93.59 ± 1.6^a^	6.41 ± 1.6^c^		DHAH	55.8 ± 1.3^bc^	52.4 ± 0.5^ab^	3.4 ± 0.9^c^	93.93 ± 1.5^a^	6.06 ± 1.5^c^
	DHAL	55 ± 1.2^bcd^	43.6 ± 0.5^d^	11.4 ± 1.3^b^	79.30 ± 2^b^	20.69 ± 2.1^b^		DHAL	55 ± 1.2^bcd^	44 ± 1.2^c^	11 ± 1.9^b^	80.03 ± 3.1^b^	19.96 ± 3.07^b^

Results are presented as mean ± SD (n = 5). Means with different superscript (a–f) letters, are significantly different from one another, according to Tukey’s multiple comparison test at *p*< 0.05. CL: comet length, HL: head length, TL: tail length, %DIH: %DNA, in head, %DIT: %DNA, in tail, NC: normal control, VC: vehicle control, PC: positive control, DC: disease control, HD: high dose, LD: low dose, ART: artemisinin, ARTEM: artemether, ARTES: artesunate, DHA: dihydroartemisinin.

### 3.10 Inflammatory pathways

**FIGURE 8:**
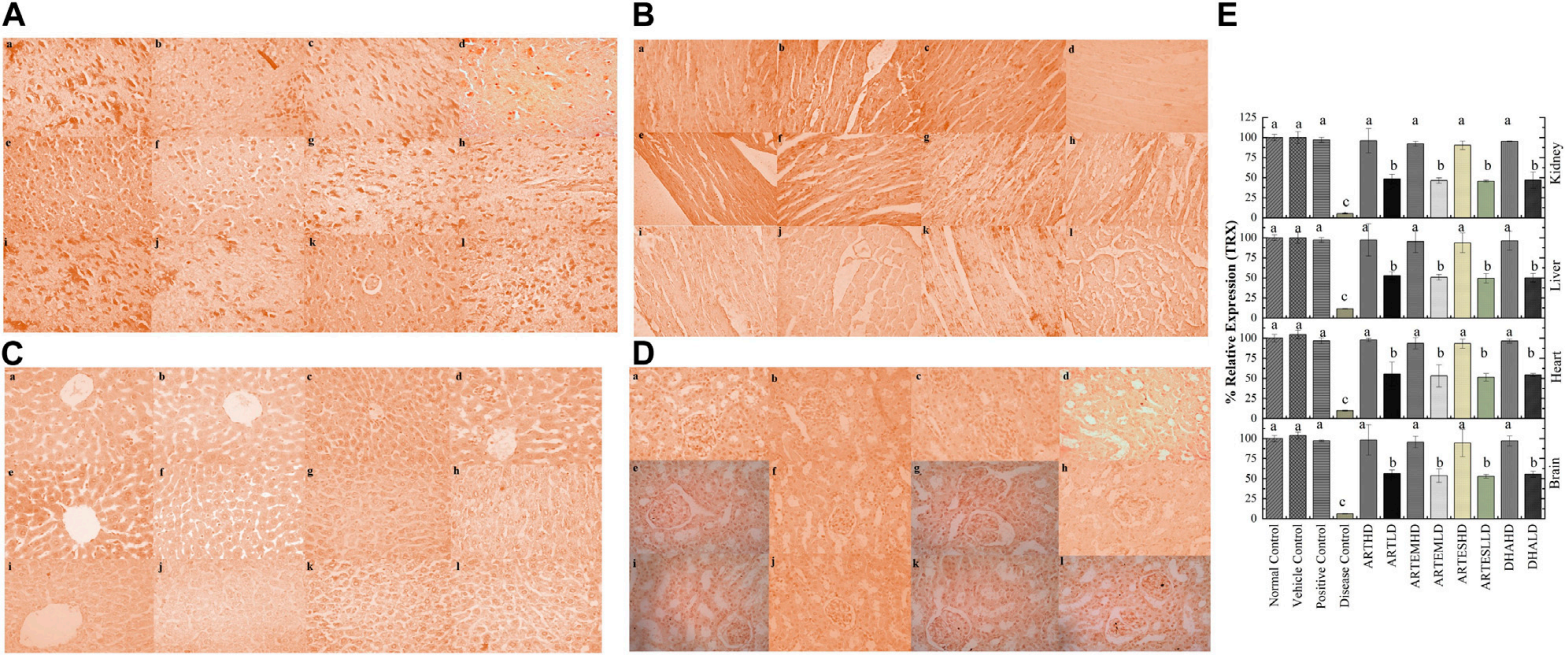
Immunohistochemical analysis of TRX in **(A)** brain, **(B)** cardiac, **(C)** hepatic, and **(D)** renal tissues. (a) Normal control, (b) vehicle control, (c) positive control, (d) disease control, (e) ARTHD, (f) ARTLD, (g) ARTEMHD, (h) ARTEMLD, (i) ARTESHD, (j) ARTESLD, (k) DHAHD, and (l) DHALD. HD, high dose; LD, low dose; ART, artemisinin; ARTEM, artemether; ARTES, artesunate; DHA, Dihydroartemisinin. **(E)** Quantitative analysis of TRX. The results are presented as mean ± SD (n = 5). Means with different superscript **(A–C)** letters are significantly different from one another, according to Tukey’s multiple comparison test at *p* < 0.05.

**FIGURE 9:**
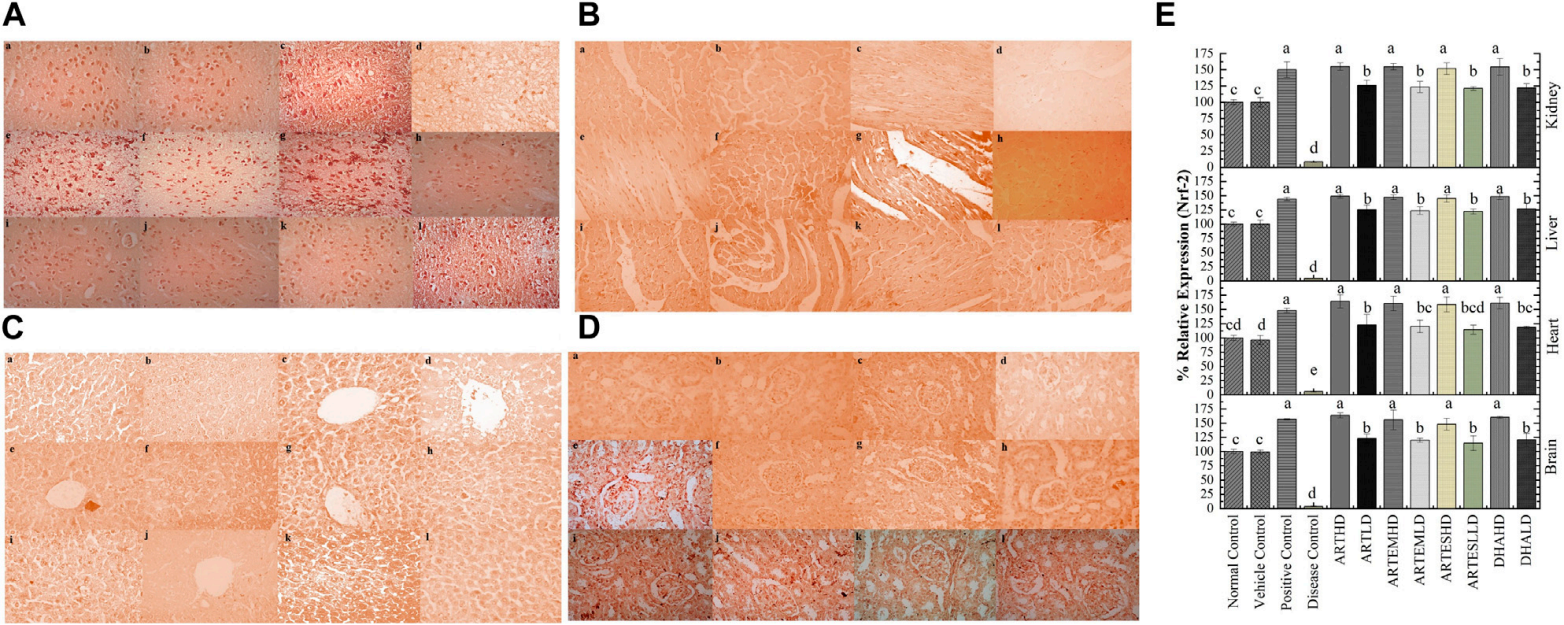
Immunohistochemical analysis of Nrf-2 in **(A)** brain, **(B)** cardiac, **(C)** hepatic, and **(D)** renal tissues. (a) Normal control, (b) vehicle control, (c) positive control, (d) disease control, (e) ARTHD, (f) ARTLD, (g) ARTEMHD, (h) ARTEMLD, (i) ARTESHD, (j) ARTESLD, (k) DHAHD, and (l) DHALD. HD, high dose; LD, low dose; ART, artemisinin; ARTEM, artemether; ARTES, artesunate; DHA, dihydroartemisinin. **(E)** Quantitative analysis of Nrf-2. The results are presented as mean ± SD (n = 5). Means with different superscript **(A–E)** letters are significantly different from one another, according to Tukey’s multiple comparison test at *p* < 0.05.

**FIGURE 10:**
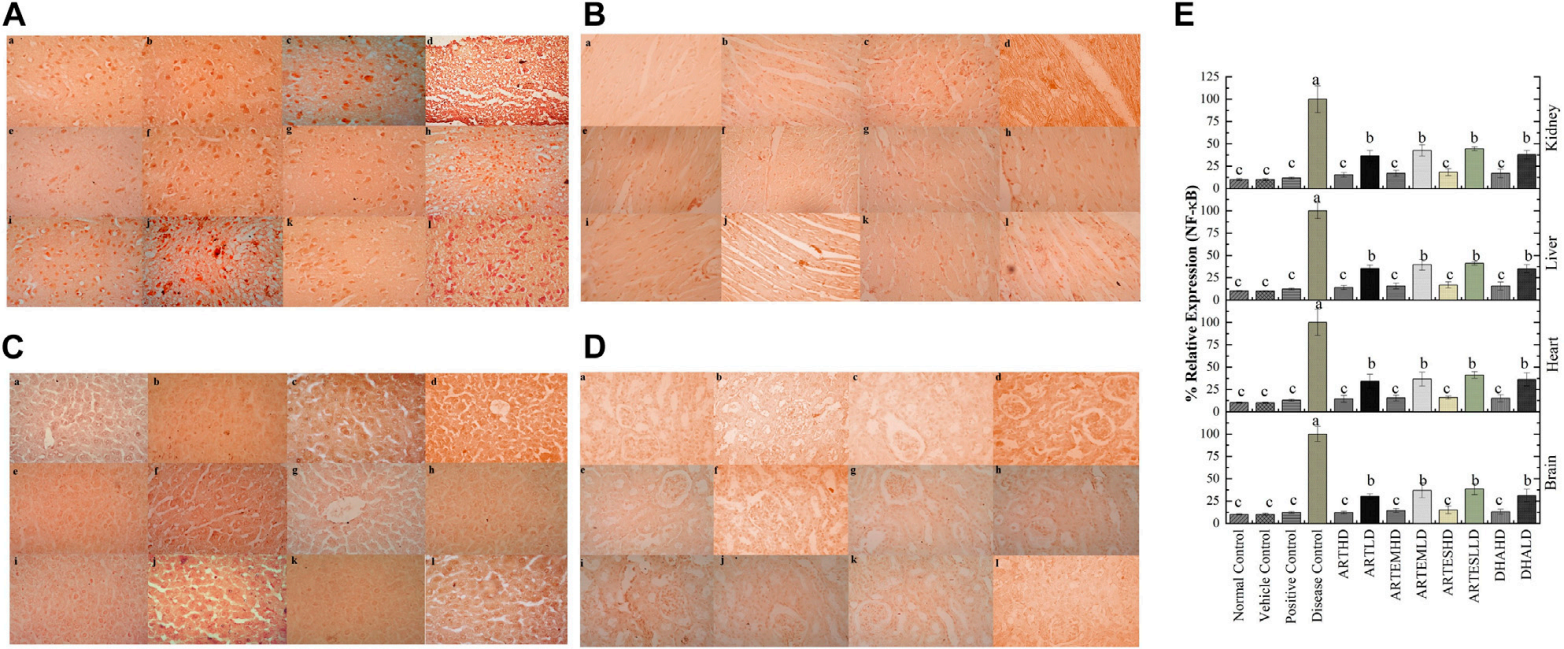
Immunohistochemical analysis of NF-κB in **(A)** brain, **(B)** cardiac, **(C)** hepatic, and **(D)** renal tissues. (a) Normal control, (b) vehicle control, (c) positive control, (d) disease control, (e) ARTHD, (f) ARTLD, (g) ARTEMHD, (h) ARTEMLD, (i) ARTESHD, (j) ARTESLD, (k) DHAHD, and (l) DHALD. HD, high dose; LD, low dose; ART, artemisinin; ARTEM, artemether; ARTES, artesunate; DHA, dihydroartemisinin. **(E)** Quantitative analysis of NF-κB. The results are presented as mean ± SD (n = 5). Means with different superscript **(A–C)** letters are significantly different from one another, according to Tukey’s multiple comparison test at *p* < 0.05.

**FIGURE 11:**
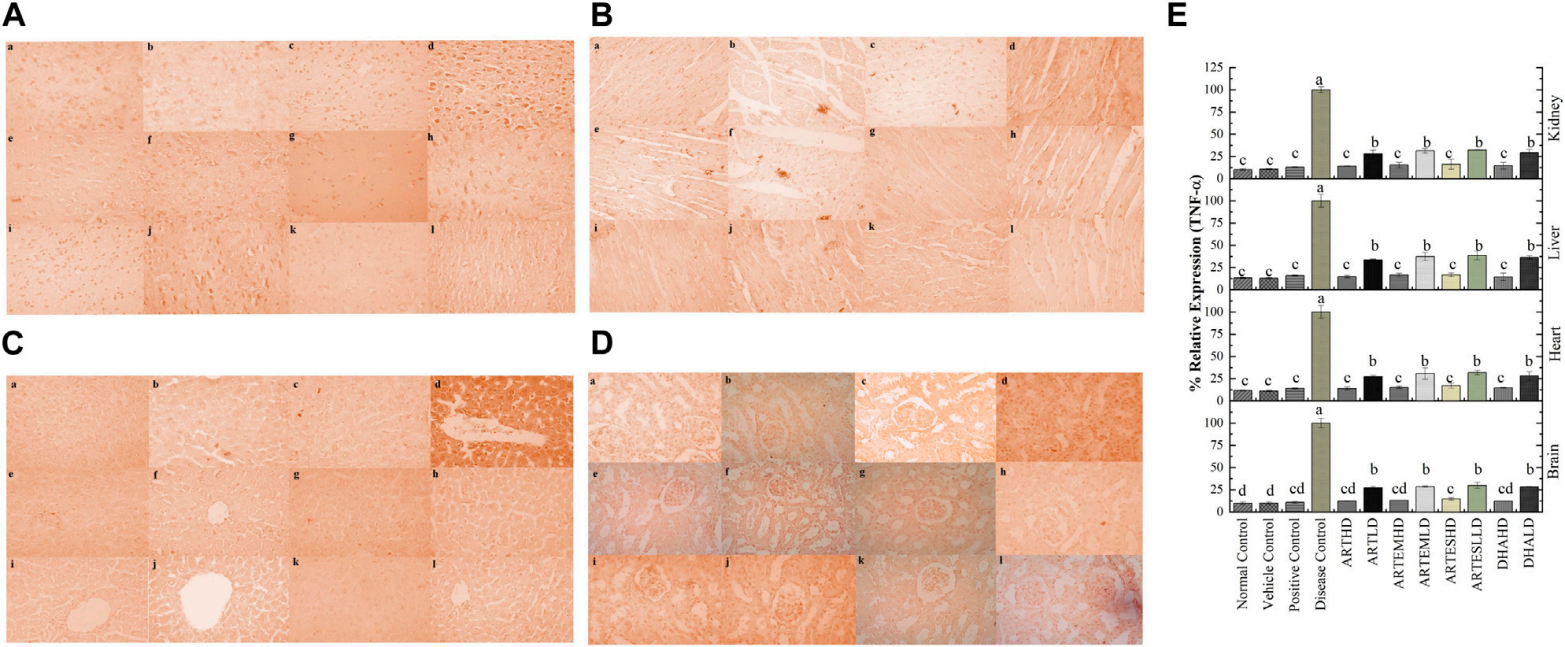
Immunohistochemical analysis of TNF-α in **(A)** brain, **(B)** cardiac, **(C)** hepatic, and **(D)** renal tissues. (a) Normal control, (b) vehicle control, (c) positive control, (d) disease control, (e) ARTHD, (f) ARTLD, (g) ARTEMHD, (h) ARTEMLD, (i) ARTESHD, (j) ARTESLD, (k) DHAHD, and (l) DHALD. HD, high dose; LD, low dose; ART, artemisinin; ARTEM, artemether; ARTES, artesunate; DHA, dihydroartemisinin. **(E)** Quantitative analysis of TNF-α. The results are presented as mean ± SD (n = 5). Means with different superscript **(A–D)** letters are significantly different from one another, according to Tukey’s multiple comparison test at *p* < 0.05.

**FIGURE 12:**
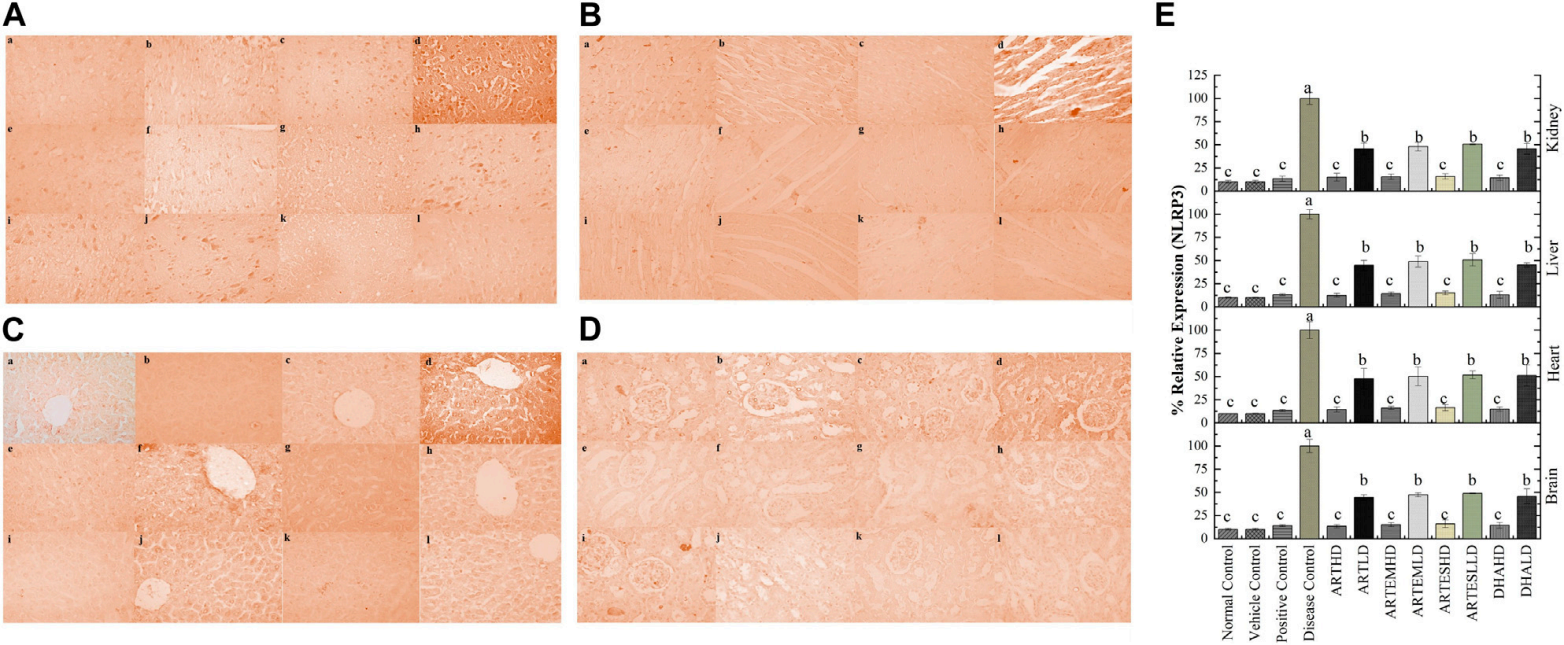
Immunohistochemical analysis of NLRP3 in **(A)** brain, **(B)** cardiac, **(C)** hepatic, and **(D)** renal tissues. (a) Normal control, (b) vehicle control, (c) positive control, (d) disease control, (e) ARTHD, (f) ARTLD, (g) ARTEMHD, (h) ARTEMLD, (i) ARTESHD, (j) ARTESLD, (k) DHAHD, and (l) DHALD. HD, high dose; LD, low dose; ART, artemisinin; ARTEM, artemether; ARTES, artesunate; DHA, dihydroartemisinin. **(E)** Quantitative analysis of NLRP3. The results are presented as mean ± SD (n = 5). Means with different superscript **(A–C)** letters are significantly different from one another, according to Tukey’s multiple comparison test at *p* < 0.05.

IHC staining of neural, cardiac, hepatic, and renal sections was performed. The results revealed that there was a significantly reduced expression of TRX, Nrf-2 and a significantly increased expression of inflammatory markers, including NF-κB, TNF-α, and NLRP3, in the disease control group. In contrast to the disease control, the AC-treated groups showed a dose-dependent augmentation in the expression of TRX and Nrf-2. Treatment with ACs also suppressed the expression of inflammatory mediators NF-κb, TNF-α, and NLRP3 (Figures 8–12). The maximum alleviation in terms of %relative expression was exhibited by ARTHD in all the tissues tested.

## 4 Discussion

Inflammation is the body’s natural defense mechanism. Short-term adaptive response is involved in initiating various tissue repair mechanisms. However, chronic inflammation possesses etiological links to several diseases. Globally, almost three of five people die because of chronic inflammatory diseases (Pahwa et al., 2022). Studies have documented a strong association between inflammation and oxidative stress. They are interconnected and interdependent processes that co-exist in the inflamed milieu (Swindle and Metcalfe, 2007).

Plant-based products are currently the subject of much research interest, with more than 200,000 bioactive compounds reported so far (Eltom at al., 2021; Baig et al., 2022a). Artemisinins, a family of sesquiterpene trioxane lactones with an endoperoxide bridge, include potent antimalarial, anticancer, and antioxidant members (Muhammad et al., 2021). These compounds also have been found to mitigate several inflammatory pathways when previously evaluated in experimental models (Yao et al., 2016). The current study was, therefore, designed for comprehensive screening and comparison of the anti-inflammatory potential of these compounds through behavioral, biochemical, histopathological, genotoxic, and immunohistochemical analysis.

Alterations in animals’ BW were measured every 2 weeks. The continuous decrease of the BW of the disease control indicates disease induction (Yoshioka et al., 2018). Artemisinic compounds significantly attenuated the BW decrease, with ARTHD exhibiting the maximum alleviation.

Inflammation imparts a more nuanced impact on social behavior (Hashioka et al., 2018). Currently, the lack of exploratory behavior and immobility observed in OFT, TST, and FST signify the anxious and depressive-like behavior of the CCl_4_-treated animals. Immobility reflects the impairment of tenacity in escape-driven behavior. Mounting evidence suggests a robust association between animal behavior and inflammatory mediators (Shi et al., 2017; Khan et al., 2019).

In the current study, the ACs showed a profound impact on anxious behavior, with ARTHD exhibiting maximum anxiolytic and antidepressant effects. It can be linked to ART’s significant potential of inhibiting the activation of currently studied inflammatory mediators, including TNF-α, NF-κB, and NLRP3. The present findings are in agreement with the previous study endorsing the antidepressant effects of artemisinic compounds (Ahangar et al., 2011).

A significant increase in %SAB (Y-maze) in the AC-treated groups indicates the overhauling of the CCl_4_-induced spatial memory deficit. Currently, the maximum improvement in cognitive and memory deficit was exhibited by ARTHD, followed by DHAHD, ARTEMHD, and ARTESHD. This potential might be attributed to their ability to reduce the amyloid-β plaque formation, which triggers the deleterious influx of calcium ions into neurons, eventually leading to cell dysfunction and death (He et al., 2021).

Oxidative stress is associated with the impaired skeletal muscle function and locomotor activity observed in the CCl_4_-treated animals (Khan et al., 2019; Lian et al., 2022). Artemisinic compounds, especially ARTHD, alleviated the weight-lifting score and time spent on the inverted screen compared to the disease control. Scientific evidence implicates inflammation’s association with cachexia. Because of their anti-inflammatory effects, artemisinic compounds might be able to restore muscular strength (Webster et al., 2020).

Hematological investigations are the prognostic tool used to assess inflammatory conditions. CCl_4_ can induce severe hemodynamic aberrations that can be linked to a concomitant rise in inflammatory cytokine levels (Nasir et al., 2022). Scientific evidence supports the notion that TNF-α directly inhibits erythropoiesis and destroys RBCs. The presence of oxygen-bound Hb within the erythrocytes makes them very prone to oxidative damage. Thrombocytopenia, increased ESR, and WBCs are established markers of chronic inflammation (Kong et al., 2021). Currently, all these perturbations can be observed in the disease control group, which is in complete accordance with a previous study (Nasir et al., 2022). Artemisinic compounds significantly diminished the CCl_4_-mediated hematological abnormalities, which can be linked to their anti-inflammatory potential (Richard, 2006). Furthermore, ART is known to restore the normal hematopoietic aptitude within the body (Fang et al., 2019).

There is ample data underscoring a causal correlation between inflammation and the seepage of cytosolic enzymes into the bloodstream. Currently, increased serum levels of CK and LDH in the disease control indicate myocardial inflammation, whereas increased ALT and AST suggest hepatic inflammation (Chen et al., 2015; Kazmi et al., 2018). Elevated levels of renal inflammatory markers, including urea, creatinine, and decreased albumin, in the disease control clearly indicate renal insufficiency (Swindle and Metcalfe, 2007; Almundarij et al., 2021). Marked differences between the findings of the disease control and AC-treated groups endorse the anti-inflammatory nature of artemisinic compounds. Herein, ARTHD was found to be more potent than other metabolites. Previously, ART has been known to reduce ALT and AST levels in people with persistent alcohol-induced liver injury (Zhao et al., 2017).

The serum was also evaluated for IL-1β levels. IL-1β is a key player in orchestrating the inflammatory response within the body. It amplifies the inflammatory response by facilitating the migration of immune cells to the site of inflammation and stimulates the production of several other pro-inflammatory mediators, such as leukotrienes and prostaglandins (Farhan et al., 2014). Currently, a surge in IL-1β levels has been observed in the disease control. Artemisinic compounds dose-dependently suppress this surge. Herein, ARTHD was found to exert pronounced anti-inflammatory effects by maximally reducing the IL-1β levels compared to the disease control. Current findings endorse a previous study where ART was found to significantly reduce IL-1β levels in mice brains (Shi et al., 2013).

Chronic inflammation may pave the way for the generation of free radicals. Nature has bestowed animals with a variety of repair endogenous antioxidants that are specific for the dismutation and detoxification of free radicals. Enzymatic antioxidants include SOD, CAT, and POD, whereas GSH is one of the major non-enzymatic antioxidants (Kazmi et al., 2018).

Exhausted antioxidant levels will foster an augmented generation of NO^•^, which induces lipid peroxidation by producing peroxynitrite (Mohammed et al., 2021). These peroxidized lipids yield TBARS, which can be used as a diagnostic index of macromolecular peroxidation, as their end products. The detrimental cycle, which initiates with a dearth of endogenous antioxidants, culminates in destroying cellular integrity and necrosis (Nasir et al., 2022).

Currently, a significant decrement in endogenous antioxidant markers with an anticipated increase in NO^•^ and TBARS levels was observed in the disease control. Treatment with artemisinic compounds, specifically with ARTHD, produced significant recuperative effects. Studies have reported that DHA and ART can exert antioxidant effects by alleviating levels of endogenous antioxidants and inhibiting inducible nitric oxide synthase (Zhao et al., 2017; Webster et al., 2020).

Histopathological analysis further authenticated the anti-inflammatory potential of artemisinic compounds. Because neurons are rich in polyunsaturated fatty acids, they are particularly sensitive to lipid peroxidation. Extensive neuronal degeneration and a relatively thinner granular layer were observed in brain sections of the disease control, which is in complete accordance with a previous study (Khan et al., 2019). Artemisinic compounds significantly enhanced hippocampal neurogenesis as evidenced by a dense granular layer with normal cells. These histological findings corroborated the results of behavioral analysis as the density of the granular layer is associated with spatial memory and learning. Moreover, antidepressants are known to accelerate neurogenesis (Altinoz et al., 2018). These accumulating pieces of evidence suggest that artemisinic compounds can treat neuroinflammation.

The histoarchitecture of cardiac slides demonstrated inflammatory cell infiltration, interstitial edema, cytoplasmic vacuolization, and widespread necrosis in the disease control. Reversion of these histological deteriorations by artemisinic compounds can be linked to concomitant inhibition of inflammatory signaling pathways, including NF-κB and NLRP3, which contribute to cardiac toxicity (Shi et al., 2017).

The hepatic tissues of the disease control exhibited fat accretion, steatosis, severe inflammation, and a distorted portal vein. The lipophilic nature of CCl_4_ and its metabolites facilitates their interaction with the lipid bilayer, eventually disrupting the cell integrity and ultrastructure (Mohammed et al., 2020; Nasir et al., 2022). A nearly normal histoarchitecture of HD-treated groups strongly substantiates the anti-inflammatory potential of artemisinic compounds.

The renal histological slides of the disease control portrayed tubular degeneration, disrupted Bowman’s capsules, glomerular congestion, and necrotic cells. Artemisinic compounds eliminated the CCl_4_-induced toxicity as smooth renal histology was portrayed by the HD-treated groups. The previously reported antifibrotic assertiveness of ART might be responsible for the observed nephroprotective potential (Dolivo et al., 2021).

A substantial body of evidence implicates increased DNA migration with DNA fragmentation. Moreover, there is a strong conviction that ROS, NO^•^, and TNF-α are embroiled in DNA strand breaks, aberrant DNA cross-linking, and mutations. The current findings agree with previous ones, where CCl_4_ exposure escalated DNA fragmentation (Khan et al., 2012). Artemisinic compounds dose-dependently reduced the CCl_4_-induced DNA toxicity. The anti-inflammatory potential of artemisinic compounds observed in the current study might be the key factor responsible for their genoprotective potential.

Inflammation interacts with an upsurge in various inflammatory mediators, cytokines, inflammasomes, and an impaired antioxidant defense system. The cytosolic transcription factor Nrf-2 is translocated into the nucleus under stress conditions, where it activates the transcription of antioxidant response element-driven genes (Bellezza et al., 2018). It targets a variety of enzymes, including proteins of the TRX system, a key redox regulatory system that can modulate the NF-κB transcription by inhibiting its nuclear translocation (Cebula et al., 2015; Yang et al., 2024). Moreover, Nrf-2 is also known for suppressing the NLRP3 and TNF-α-dependent NF-κB pathways (Wakabayashi et al., 2010; Jhang and Yen, 2017).

The redox-regulated transcription factor NF-κB is involved in the regulation of inflammatory responses by the induction of pro-inflammatory cytokines, including TNF-α (Jalal et al., 2022). The inflammasome NLRP3 is also involved in the processing and release of several pro-inflammatory cytokines. Its activation generally requires a TNF-α-triggered priming process via NF-κB-dependent pathways. TNF-α is a cytokine that has been identified as a major regulator of inflammatory responses (Bauernfeind et al., 2009; Ahsan et al., 2023). CCl_4_ induces inflammation by modulating these interlinked signaling pathways (Khan et al., 2019). In the current study, artemisinic compounds ameliorated inflammation by activating the Nrf-2 and TRX signaling pathways compared to the disease control. Our experimental data also revealed the inhibitory effect of artemisinic compounds on the activation of NF-κB and NLRP3, thus leading to the decreased production of the downstream cytokines TNFa and IL-1b. The results produced by the ACs, especially ARTHD, were comparable with those observed in the silymarin-treated group. The vehicle control did not exhibit any toxicity or activity throughout the study, suggesting its neutral nature.

The maximum anti-inflammatory potential was exhibited by ART, followed by DHA, ARTEM, and ARTES. Alkylating center reactivity, side chain, lipophilicity, molecular geometry, and electronic features are parameters that may be responsible for the biological activities of sesquiterpene lactones (Ghantous et al., 2010). Facile hydrolysis of the ARTES ester linkage and cytochrome P450-mediated hepatic metabolism of ARTEM allows their conversion to the highly active metabolite, DHA. DHA, a reduced product of ART, is very prone to oxidative attacks owing to the lactol moiety present in its structure. Its hemiacetal structure gives it stability compared to ART (Aprioku and Obianime, 2011). Metabolism of ART generates six major metabolites, including monohydroxylated deoxyART, deoxy ART, monohydroxylated ART, dihydroxylated ART, ART, 9,10-dihydrodeoxyartemisinin, and a metabolite named “crystal 7” (Zang et al., 2014). Hydroxylated metabolites are considered biologically more active as they are reported to enhance water solubility and binding affinity to the targets (Gao et al., 2023). Deoxy metabolites, such as deoxyART, a phase-I metabolite of ART metabolism, are known to possess anti-inflammatory activity. Moreover, ART and DHA share a common metabolite, deoxyDHA (Fu et al., 2020). Therefore, it can be inferred that metabolites of ART and DHA are responsible for the superlative anti-inflammatory potential of ACs.

Current findings suggest that artemisinic compounds can serve as a solid alternative to existing therapeutics. Modulating oxidative stress pathways (Nrf2/TRX) and inhibiting inflammatory mediators (NFκB/TNF-α/NLRP3) facilitates them in managing various inflammatory conditions. A protective behavior toward DNA depicted by comet assay and toward the organs tested by histological analysis added more to the safety profile of ACs. Additionally, the reduced risk of drug resistance and low toxicity profile offer advantages over conventional anti-inflammatory drugs with several adverse effects, including immune suppression. Studies have indicated the adverse effects of NSAIDs in cardiovascular, cerebral, pulmonary, gastrointestinal, hepatic, and renal complications (Bindu et al., 2020).

## 5 Conclusion

The cumulative findings of all the behavioral, biochemical, histological, genotoxic, and IHC analyses provide convincing evidence that artemisinic compounds possess significant anti-inflammatory potential. The anxiolytic and antidepressant potential of artemisinic compounds indicates suppression of neuroinflammation, whereas serum analysis indicates the inhibition of cardiac, hepatic, and renal inflammation. Revitalization of endogenous antioxidants and suppression of inflammatory markers also support this finding. Moreover, artemisinic compounds were found to be non-toxic to DNA. Among the selected ACs, ART was found to be the most efficacious. The current study serves as a cornerstone to establish the fundamental framework for more in-depth molecular investigations that can elucidate the structure–activity relationship of artemisinic compounds with their anti-inflammatory potential. The exploration of the AIP of artemisinic compounds presents promising avenues for treating diverse inflammatory disorders by targeting pivotal pathways. The broad spectrum of activity, proven efficacy, and global availability of APs could allow them to effectively alleviate several inflammation-associated pathologies, including autoimmune disorders, neuroinflammatory diseases, cardiovascular conditions, cancer, and many others. The current study accomplished the repurposing of artemisinic compounds, renowned for their antimalarial potential, as a potential treatment for inflammatory diseases.

## Data Availability

The raw data supporting the conclusion of this article will be made available by the authors, without undue reservation.

## Abbreviations

ART, artemisinin; ARTEM, artemether; ARTES, artesunate; CCl4, carbon tetrachloride; DHA, dihydroartemisinin; IHC, immunohistochemistry; IL-1β, interleukin-1beta; NF-κB, nuclear factor kappa light chain enhancer of activated B cells; NLRP3, nucleotide-binding oligomerization domain-like receptors protein 3; Nrf-2, nuclear factor erythroid-2- related factor; ROS, reactive oxygen species; TNF-α, tumor necrosis factor alpha; TRX, thioredoxin.
